# Patient-Specific TBX5-G125R Variant Induces Profound Transcriptional Deregulation and Atrial Dysfunction

**DOI:** 10.1161/CIRCULATIONAHA.121.054347

**Published:** 2022-02-03

**Authors:** Antoinette F. van Ouwerkerk, Fernanda M. Bosada, Karel van Duijvenboden, Arjan C. Houweling, Koen T. Scholman, Vincent Wakker, Cornelis P. Allaart, Jae-Sun Uhm, Inge B. Mathijssen, Ton Baartscheer, Alex V. Postma, Phil Barnett, Arie O. Verkerk, Bastiaan J. Boukens, Vincent M. Christoffels

**Affiliations:** Department of Medical Biology (A.F.vO., F.M.B., K.vD., K.T.S., V.W., J-S.U., A.V.P., P.B., A.O.V., B.J.B., V.M.C.), University of Amsterdam, The Netherlands.; Experimental Cardiology (T.B., A.O.V.), University of Amsterdam, The Netherlands.; Department of Cardiology, Vrije Universiteit Amsterdam (C.P.A.), Amsterdam Cardiovascular Sciences, The Netherlands.; Department of Human Genetics (A.C.H., I.B.M., A.V.P.), Amsterdam University Medical Center, The Netherlands.; Aix-Marseille University, INSERM, TAGC, Marseille, France (A.F.vO.).

**Keywords:** arrhythmias, cardiac, epigenesis, genetic, myocytes, cardiac, sequence analysis, RNA, T-box transcription factor 5, transcription factors

## Abstract

The pathogenic missense variant p.G125R in TBX5 (T-box transcription factor 5) causes Holt–Oram syndrome (also known as hand–heart syndrome) and early onset of atrial fibrillation. Revealing how an altered key developmental transcription factor modulates cardiac physiology in vivo will provide unique insights into the mechanisms underlying atrial fibrillation in these patients.

**Methods:**

We analyzed ECGs of an extended family pedigree of Holt–Oram syndrome patients. Next, we introduced the TBX5-p.G125R variant in the mouse genome (*Tbx5*^*G125R*^) and performed electrophysiologic analyses (ECG, optical mapping, patch clamp, intracellular calcium measurements), transcriptomics (single-nuclei and tissue RNA sequencing), and epigenetic profiling (assay for transposase-accessible chromatin using sequencing, H3K27ac [histone H3 lysine 27 acetylation] CUT&RUN [cleavage under targets and release under nuclease sequencing]).

**Results:**

We discovered high incidence of atrial extra systoles and atrioventricular conduction disturbances in Holt–Oram syndrome patients. *Tbx5*^*G125R/+*^ mice were morphologically unaffected and displayed variable RR intervals, atrial extra systoles, and susceptibility to atrial fibrillation, reminiscent of TBX5-p.G125R patients. Atrial conduction velocity was not affected but systolic and diastolic intracellular calcium concentrations were decreased and action potentials were prolonged in isolated cardiomyocytes of *Tbx5*^*G125R/+*^ mice compared with controls. Transcriptional profiling of atria revealed the most profound transcriptional changes in cardiomyocytes versus other cell types, and identified over a thousand coding and noncoding transcripts that were differentially expressed. Epigenetic profiling uncovered thousands of TBX5-p.G125R-sensitive, putative regulatory elements (including enhancers) that gained accessibility in atrial cardiomyocytes. The majority of sites with increased accessibility were occupied by Tbx5. The small group of sites with reduced accessibility was enriched for DNA-binding motifs of members of the SP (specificity protein) and KLF (Krüppel-like factor) families of transcription factors. These data show that Tbx5-p.G125R induces changes in regulatory element activity, alters transcriptional regulation, and changes cardiomyocyte behavior, possibly caused by altered DNA binding and cooperativity properties.

**Conclusions:**

Our data reveal that a disease-causing missense variant in TBX5 induces profound changes in the atrial transcriptional regulatory network and epigenetic state in vivo, leading to arrhythmia reminiscent of those seen in human TBX5-p.G125R variant carriers.

## Abstract 2

Supplemental Digital Content is available in the text.

**Clinical Perspective**

**What Is New?**

- A pathogenic variant in the fifth exon of TBX5 ([T-box transcription factor 5] p.G125R) found in a Dutch, atypical Holt–Oram syndrome family with early onset atrial fibrillation was modeled in mice.
- This is the first human pathogenic variant based on a patient family in this key cardiac transcription factor explored in vivo.
- We identify widespread electrophysiologic, transcriptional, and epigenetic changes (including coding and noncoding RNA, chromatin accessibility, and H3K27ac [histone H3 lysine 27 acetylation] association) in the atria of Tbx5-p.G125R heterozygous mice distinct from the changes in atria of Tbx5 insufficiency models.

**What Are the Clinical Implications?**

- The characterization of the Tbx5 p.G125R mouse model indicates that a patient-specific pathogenic variant in TBX5 induces changes in regulatory element activity, altered balance in the regulatory network of atrial cardiomyocytes, and clinically relevant changes in cardiomyocyte function.
- These findings shed light on the target and off-target genes of Tbx5-p.G125R because of its altered properties, which are at the basis of distinct electrophysiologic changes observed in a subset of Holt–Oram syndrome patients with atrial fibrillation.
- This work may provide insight into the epigenetic changes and transcriptional underpinning of arrhythmia in the general population caused by small increases in TBX5-expression caused by common variants predisposing to atrial fibrillation.

Pathogenic variants in T-box transcription factor (TF) *TBX5* (T-box transcription factor 5) have been linked to Holt–Oram syndrome (HOS), which is typically characterized by varying degrees of limb and cardiac malformations and conduction defects (OMIM [Online Mendelian Inheritance in Man] #142900).^1–3^ Tbx5 is dose-dependently required for heart development and specification and function of the conduction system, and determines the working myocardial phenotype of the atrium.^3–8^ Tbx5 has been put forward as key regulator of ion-handling protein-encoding gene expression and rhythm control in vivo.^9–13^ Genome-wide association studies uncovered several variants near *TBX5* associated with atrial fibrillation (AF). Moreover, transcriptome-wide analysis of human heart tissue identified an association of increased *TBX5* expression with AF risk.^14^

A pathogenic variant in the fifth exon of *TBX5* causing a glycine to arginine substitution (c.373G>A; p.G125R) in the T-box domain was found in a Dutch, atypical HOS family.^15^ In this family, affected members show HOS symptoms such as mild skeletal abnormalities and septal defects, as well as atypical early onset paroxysmal AF. The function of missense variants in this key TF and the impact on gene regulation in the context of heart function in vivo are poorly understood. Therefore, we engineered the p.G125R variant in the highly conserved T-box of mouse *Tbx5*, and used this model to gain insight into the molecular mechanisms underlying structural and electrophysiological changes predisposing to AF in *TBX5* missense variant carriers.

## Methods

Detailed Methods are provided in the Supplemental Material.

### Data Availability

Whole-tissue RNA-sequencing ([RNA-seq] left and right atrial), cardiomyocyte (CM) nucleus H3K27ac (histone H3 lysine 27 acetylation) CUT&RUN (cleavage under targets and release under nuclease), CM nucleus assay for transposase-accessible chromatin using sequencing (ATAC-seq), and single-nuclei RNA-seq are available on Gene Expression Omnibus (GSE167082). All other data and supporting materials have been provided with the published article.

### Informed Consent Statement

The study was conducted according to the guidelines of the Declaration of Helsinki. Written informed consent was obtained from the families presented in the paper.

### Animal Care

Animal care and experiments conform to the Directive 2010/63/EU of the European Parliament. All animal work was approved by the Animal Experimental Committee of the Amsterdam University Medical Centers (location Academic Medical Center, Amsterdam, the Netherlands), was performed in compliance with the Dutch government guidelines, and was approved by the Central Committee Animal Experiments.

### Statistics

A detailed description of the statistics can be found in the Supplemental Material which also describes the specific statistical tests used to analyze large data sets. Results of statistical analysis are given in the text or the figure legends.

## Results

### Mouse Model Recapitulates Atrial Arrhythmia and ECG Abnormalities in TBX5-G125R Patients

**Figure 1.**
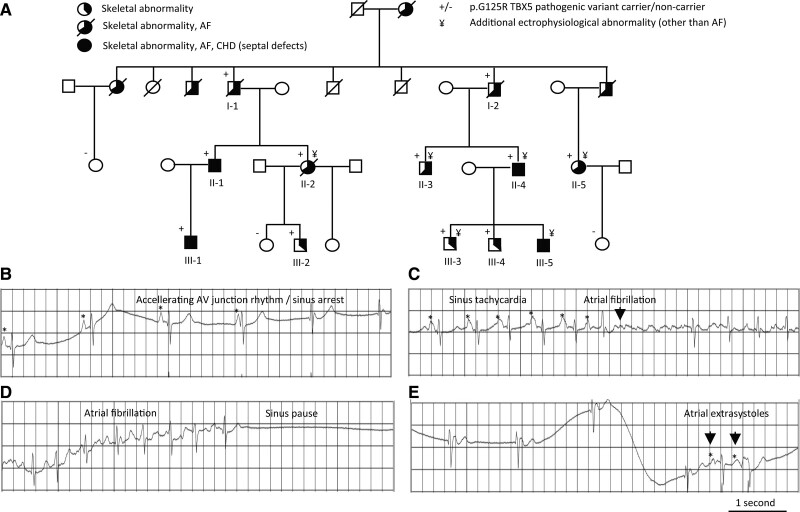
**Pedigree and electrophysiological abnormalities found in a family with atypical Holt–Oram syndrome with early onset AF.** **A**, Pedigree showing the presence of skeletal abnormalities, congenital heart disease (ie, septal defects) and AF in TBX5p.G125R carriers. **B** through **E**, Example ECG traces of electrophysiological abnormalities found within 1 individual with the TBX5p.G125R variant (Patient II-5; Table S1). AF indicates atrial fibrillation; AV, atrioventricular; CHD, congenital heart disease; and TBX5, T-box transcription factor 5.

Heterozygous TBX5-p.G125R carriers show atypical HOS, including cardiac septal defects, mild skeletal abnormalities, and early onset AF (Figure 1A).^15^ Here, we reanalyzed in detail the available ECG traces (as obtained by 24-h Holter or 12-lead ECG) of the patients previously analyzed.^15^ We found a range of additional abnormalities—including irregular RR interval, right bundle branch block, atrioventricular (AV) junctional escape, AV junctional rhythm, sinus arrest, left ventricular noncompaction, atrial extras, and sick sinus syndrome—that were not checked for previously (Figure 1B–1E; Table S1). This shows that these patients suffer from other types of supraventricular arrhythmia that indicate dysfunctionality of the atrial muscle, including the sinoatrial and AV nodes.

To investigate how the p.G125R pathogenic variant in TBX5 affects heart function, we generated a mouse model with this point mutation in *Tbx5* using CRISPR-Cas9 (clustered regularly interspaced short palindromic repeats–CRISPR-associated protein 9). Interbreeding of *Tbx5*^*G125R/+*^ animals showed that from embryonic day (E) 12.5 to E16.5, homozygous *Tbx5*^*G125R/G125R*^ fetuses were necrotic or dead (Table S2), revealing that homozygosity of the mutation is incompatible with life. *Tbx5*^*G125R/G125R*^ fetuses showed ventricular and atrial septal defects (E14.5–E16.5), as well as enlarged right atria at E16.5 (Figure S1), which were also present in patients heterozygous for this pathogenic variant (Figure 1A; Table S1). We did not observe differences in CM proliferation rates in embryonic (E12.5) right atria or left ventricles between different genotypes (Figure S2), nor differences in the heart weight–tibia length ratio between control (n=6) and *Tbx5*^*G125R/+*^ mice (n=10) at 20 days after birth (Table S3).

**Figure 2.**
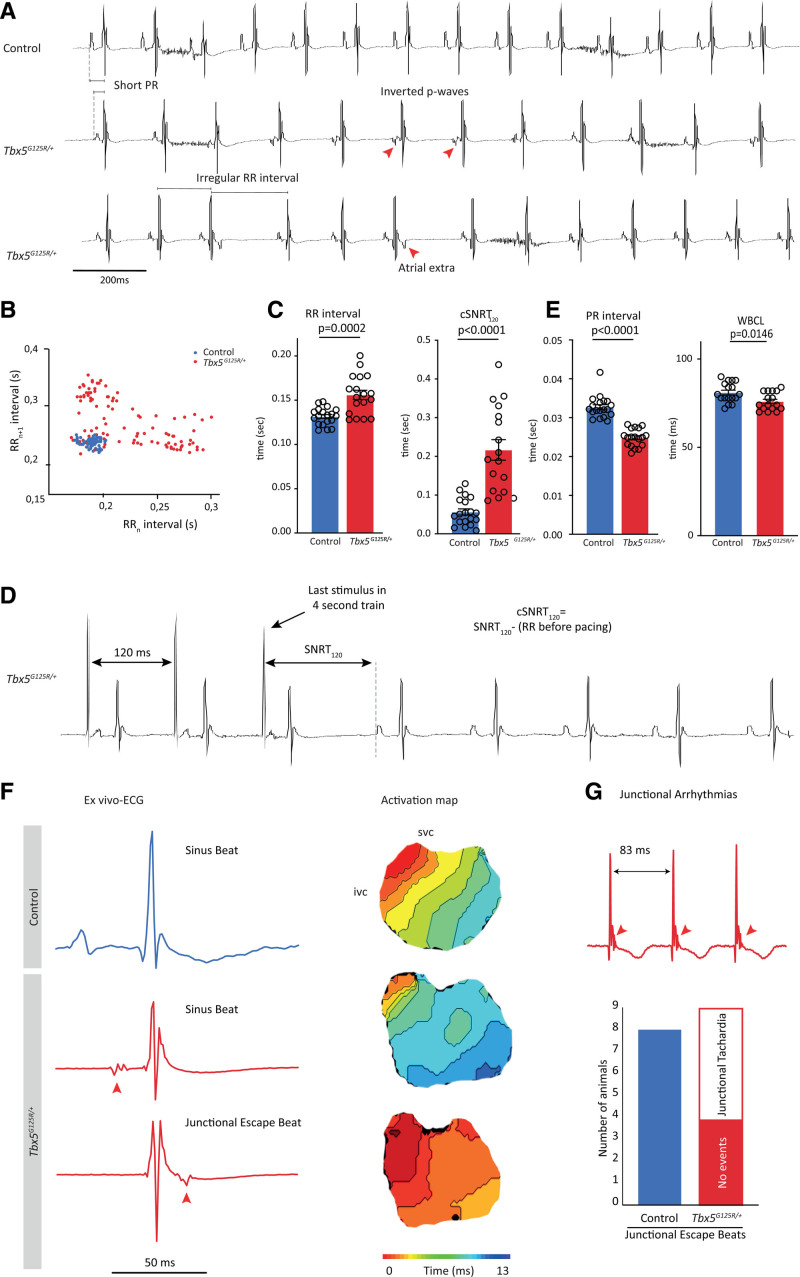
**Transesophageal burst pacing (TEBP) of Tbx5G125R/+ mice shows atrial premature activation, shortened PR interval and ventricular premature activation.** **A**, Typical example ECG traces of controls (n=19) and *Tbx5*^*G125R/+*^ mice (n=18). **B**, Scatterplot illustrating the beat-to-beat variability in RR interval of 1 control (blue) and 1 *Tbx5*^*G125R/+*^ mouse (red). **C**, Significant changes were observed in RR interval, RRsd (heart rate variability RRsd=SD of RR_n_–RR_n+1_), and SNRT of *Tbx5*^*G125R/+*^ mice (red). Genotypes were compared using Mann–Whitney *U* test; *P* values are given in the graphs. **D**, Typical example trace showing the analysis of cSNRT_120_. **E**, PR interval and WBCL are significantly shortened in *Tbx5*^*G125R/+*^ mice. Genotypes were compared using Mann–Whitney *U* test. **F**, Ex vivo typical example ECGs and corresponding activation maps (optical mapping) show atrial ectopic beats occurring during or after the QRS complex in response to a premature ventricular beat that originates in the atrioventricular junction in *Tbx5*^*G125R/+*^ mice. Red arrows indicate inverted premature beat (P-top). **G**, Junctional escape beats, not observed in 8 control mice, turn into junctional tachycardia in 55% (5/9) *Tbx5*^*G125R/+*^ mice. cSNRT_120_ indicates cyclic sinus node recovery time after 120-ms interval stimulation; ivc, inferior vena cava; SNRT, sinus node recovery time; svc, superior vena cava; TBX5, T-box transcription factor 5; and WBCL, Wenckebach cycle length.

To determine the impact of Tbx5-p.G125R on electrophysiologic function, we recorded ECGs from male and female control mice (n=19) and *Tbx5*^*G125R/+*^ mice (n=18; Figure 2A). While QRS duration was unchanged (data not shown), the *Tbx5*^*G125R/+*^ mice showed longer and more variable RR intervals than controls (Figure 2B and 2C). In addition, the sinus node recovery time measured during transesophageal pacing was prolonged in *Tbx5*^*G125R/+*^ mice (Figure 2C and 2D). This presumably sinus nodal phenotype of the *Tbx5*^*G125R/+*^ mice was accompanied by dysfunction of the AV junction as evidenced by shorter PR intervals (difference between means ± SEM, 0.007 ± 0.001 s [males]; 0.011 ± 0.002 s [females]) and Wenckebach cycle lengths compared with controls (Figure 2E). Moreover, we found atrial ectopic beats with inverted P-waves in 6 of 18 animals (Figure 2A), which explains the variable RR intervals observed in *Tbx5*^*G125R/+*^ mice. To investigate the origin of these ectopic beats we recorded optical action potentials from isolated Langendorff-perfused hearts (Figure S3A). Hearts isolated from *Tbx5*^*G125R/+*^ mice also showed longer and more variable RR intervals, as well as shorter PR intervals, than controls (Figure S3B and S3C). In addition, these experiments show that the atrial ectopic beats occurred during or after the QRS complex, and occurred in all cases in response to a premature ventricular beat (Figure 2F). The morphology of the QRS complexes of these premature beats were similar, indicating that the origin was in the AV junction and that it caused retrograde activation of the atria. The junctional escape beats were present in 100% of *Tbx5*^*G125R/+*^ mice and turned into junctional tachycardia in 60% of the cases (Figure 2G).

**Figure 3.**
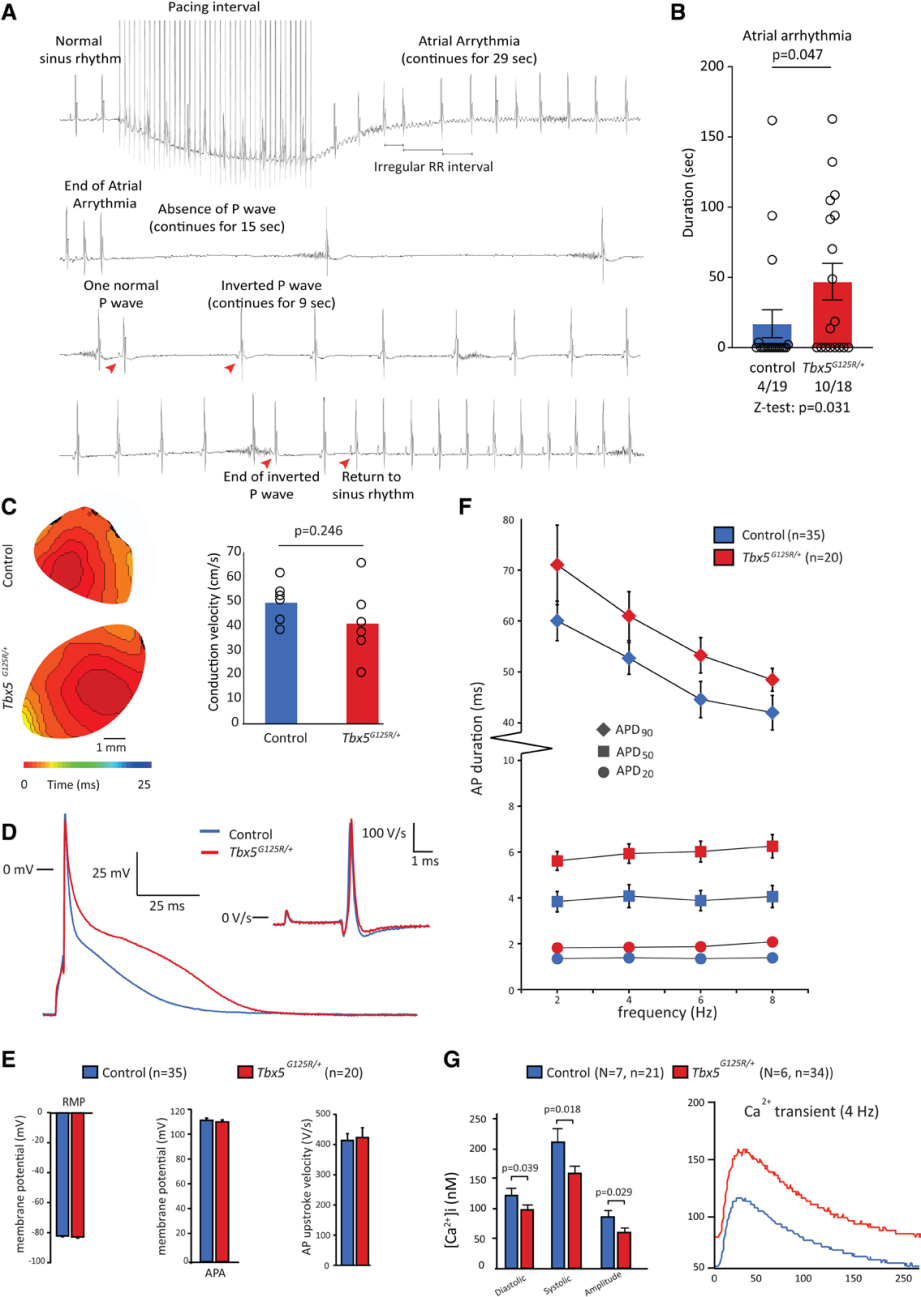
**Conduction velocity, cellular electrophysiology, and intracellular Ca2+ measurements of control and Tbx5G125R/+ mice.** **A**, Typical ECG trace of atrial arrhythmia in *Tbx5*^*G125R/+*^ mice. **B**, Transesophageal burst pacing shows more *Tbx5*^*G125R/+*^ mice with atrial arrhythmia >1 s compared with controls. Atrial arrhythmia occurred significantly more often in *Tbx5*^*G125R/+*^ mice (Z-test) and, when present, atrial arrhythmia duration was longer in these mice (Mann–Whitney *U* test). **C**, Conduction velocity does not differ between control and *Tbx5*^*G125R/+*^ mice (Mann–Whitney *U* test). **D**, Typical action potentials at 6 Hz of isolated cardiomyocytes. **E**, No significant between-genotype differences were observed for average resting membrane potential, action potential amplitude, and action potential upstroke velocity using an independent sample *t* test. **F**, APD at 20%, 50%, and 90% of repolarization (APD_20_, APD_50_, APD_90_, respectively) was tested using 3-way ANOVA, showing significant interactions of genotype and frequency with APD group, followed by 2-way ANOVA per repolarization group. These 2-way ANOVAs showed that APD differs between genotypes independently of frequency in APD_20_ (frequency: *P*=0.302, genotype: *P* < 0.001) and APD_50_ (frequency: *P*=0.459; genotype: *P* < 0.001). In APD_90_, there is both a frequency (*P* < 0.001) and a genotype (*P* < 0.001) effect but this genotype effect is independent of the frequency (interaction: *P*=0.766). **G**, At a frequency of 8 Hz, significantly lower Ca^2+^ transient amplitudes, as well as a decreased diastolic and systolic Ca^2+^ concentrations, were observed in *Tbx5*^*G125R/+*^ mice (Mann–Whitney *U* test; performed on cells per group). On the right, typical Ca^2+^ transient amplitudes example measured at 4 Hz are shown. APD indicates action potential duration; and *Tbx5*, T-box transcription factor 5.

To assess whether *Tbx5*^*G125R/+*^ mice were susceptible to atrial arrhythmias, we performed programmed atrial stimulation in vivo using a transesophageal catheter (Figure 3A). We were able to induce atrial arrhythmias in 10 of 19 *Tbx5*^*G125R/+*^ mice and 4 of 18 control mice (duration of AA >10 s; Figure 3B). We optically measured conduction velocity in the atrium of isolated hearts, which was not different between genotypes (Figure 3C; Table S4). Similarly, recorded action potentials in isolated atrial CMs (Figure 3D) revealed no difference in resting membrane potential, action potential amplitude, or upstroke velocity (Figure 3E). Action potential duration, on the other hand, was longer in atrial myocytes from *Tbx5*^*G125R/+*^ at all frequencies tested (2, 4, 6, and 8 Hz) and at all action potential repolarization phases (ie, action potential duration [APD] measured at 20%, 50%, and 90% of repolarization; Figure 3F). Intracellular Ca^2+^ measurements revealed that Ca^2+^ transients had lower amplitudes in *Tbx5*^*G125R/+*^ mice, as well as decreased diastolic and systolic Ca^2+^ concentrations, while the decay of the Ca^2+^ transient was unaffected (Figure 3G; Table S5).

### Deregulated Gene Expression in Atrial CMs of Tbx5^G125R/+^ Mice

**Figure 4.**
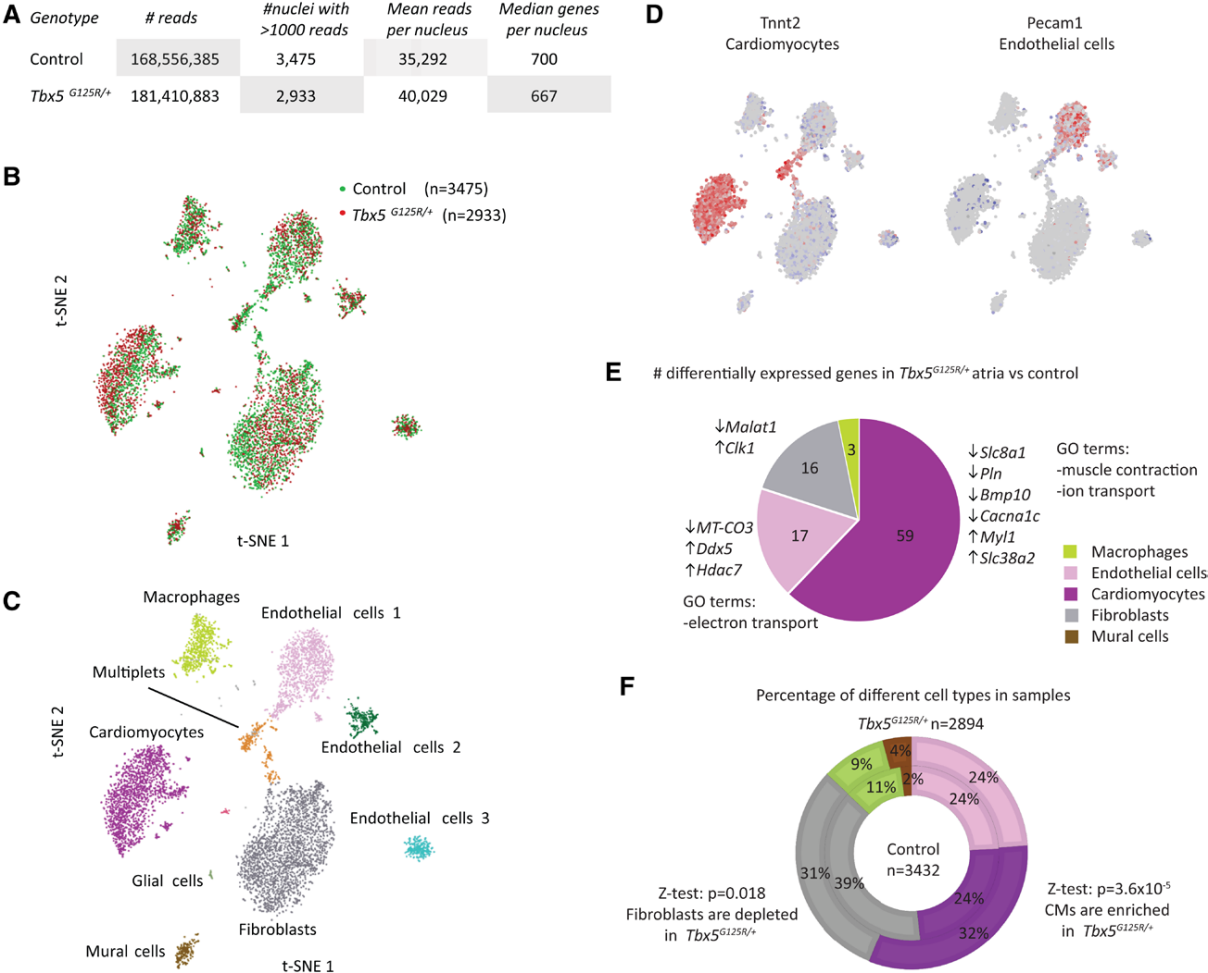
**Single-nucleus transcriptomics of control and Tbx5G125R/+ atria.** **A**, Number of reads and nuclei, mean reads, and mean genes per nucleus for control and *Tbx5*^*G125R/+*^ sample. **B**, tSNE map of control and *Tbx5*^*G125R/+*^ clusters. **C**, tSNE map showing different cell-type clusters. **D**, tSNE maps showing expression of different marker genes. **E**, Distribution of differential expression in different cell types (upregulated in *Tbx5*^*G125R/+*^ vs control ↑, and downregulated ↓). Significance of differential gene expression in single-nucleus RNA sequencing was determined by Mann–Whitney *U* test in the *R*^2^ genomics analysis and visualization platform (http://r2.amc.nl). **F**, Contribution of different cell types to control and *Tbx5*^*G125R/+*^ samples showing increase in CMs and decrease in fibroblast contribution in *Tbx5*^*G125R/+*^ (Z-test was performed to test the significance of the difference between genotypes of the fractions of cell types). *Bmp10* indicates bone morphogenetic protein 10; CM, cardiomyocyte; *Clk1*, CDC Like kinase 1; *Cacna1c*, calcium voltage-gated channel subunit α1C; *Ddx5*, DEAD-Box Helicase 5; GO, Gene Ontology; *Hdac7*, histone deacetylase 7; *Malat1*, Metastasis Associated Lung Adenocarcinoma Transcript 1; *Myl1*, Myosin Light Chain 1; *MT*-*CO3*, XXX; Pecam1, Platelet And Endothelial Cell Adhesion Molecule 1; *Pln*, phospholamban; *Slc8a1*; solute carrier family 8 member A1; *Slc38a2*, solute carrier family 38 member A2; t-SNE, T-distributed stochastic neighbor embedding; *Tbx5*, T-box transcription factor 5; and Tnnt2, Troponin T2, Cardiac Type.

To gain insight into the cell types affected by Tbx5-p.G125R, we performed single-nucleus RNA-seq on right atrial tissue. Profiles from 3475 wild-type nuclei and 2933 *Tbx5*^*G125R/+*^ nuclei were obtained (Figure 4A). T-distributed stochastic neighbor embedding was used to identify 9 clusters (Figure 4B), corresponding to cardiac cell types based on marker gene expression (Figure 4C and 4D; Figure S4). Only the CM cluster showed some degree of segregation between genotypes (Figure 4B). We determined differential expression within each cluster by Kruskal–Wallis test (Bonferroni corrected *P* < 0.05). CMs showed 59 genes that were significantly differentially expressed; endothelial cells showed 17 genes and fibroblasts showed 16 (Figure 4E; Table S6). Analyses of the Gene Ontology terms for the differentially expressed genes in CMs showed mostly terms involving muscle contraction regulation and ion transport (Table S7). The fraction of CMs and fibroblasts differed between genotypes (CMs enriched in *Tbx5*^*G125R/+*^ [936/2894] vs controls [828/3432]; Z-test, *P*=3.68×10^-5^; fibroblasts depleted in *Tbx5*^*G125R/+*^ [255/2894] vs controls [363/3432]; Z-test, *P*=0.018; Figure 4F). Immunofluorescence analysis revealed ratios of Nkx2-5+ (NK2 homeobox 5) CM nuclei over non-CM nuclei were significantly higher in atria of 8-month-old *Tbx5*^*G125R/+*^ mice compared with controls (*P*=0.014; Figure S5A). Picrosirius red–staining sections of 8-month-old control (n=4) and *Tbx5*^*G125R/+*^ (n=5) atria revealed a trend toward a smaller fibrotic area in the atria of *Tbx5*^*G125R/+*^ mice compared with controls; however, it was not significant (Figure S5B and 5C). Together, these findings suggest that the *Tbx5*^*G125R/+*^ atria do not show elevated fibrosis even though fibrosis has been associated with AF.

To determine at greater depth the transcriptional responses to heterozygosity of Tbx5-p.G125R, RNA-seq was performed on whole right atria of adult female control (n=5) and *Tbx5*^*G125R/+*^ (n=5) animals (Figure S6A). The point mutation may affect mRNA levels of *Tbx5*^*G125R*^ (ie, nonsense-mediated decay). Using RNA-seq reads and Varscan,^16^ we quantified the number of *Tbx5* transcripts tags containing the wild-type allele (G) and the number containing the mutant allele (C). In control animals, the wild-type allele had a frequency of 100%. In *Tbx5*^*G125R/+*^ animals, the variant allele had a frequency of 54% (202 wild-type [C] vs 242 mutant [G] tags; Fisher exact test, *P*=0.98), indicating both the wild-type and *Tbx5*^*G125R/+*^ transcripts are present at equal levels in *Tbx5*^*G125R/+*^ animals.

**Figure 5.**
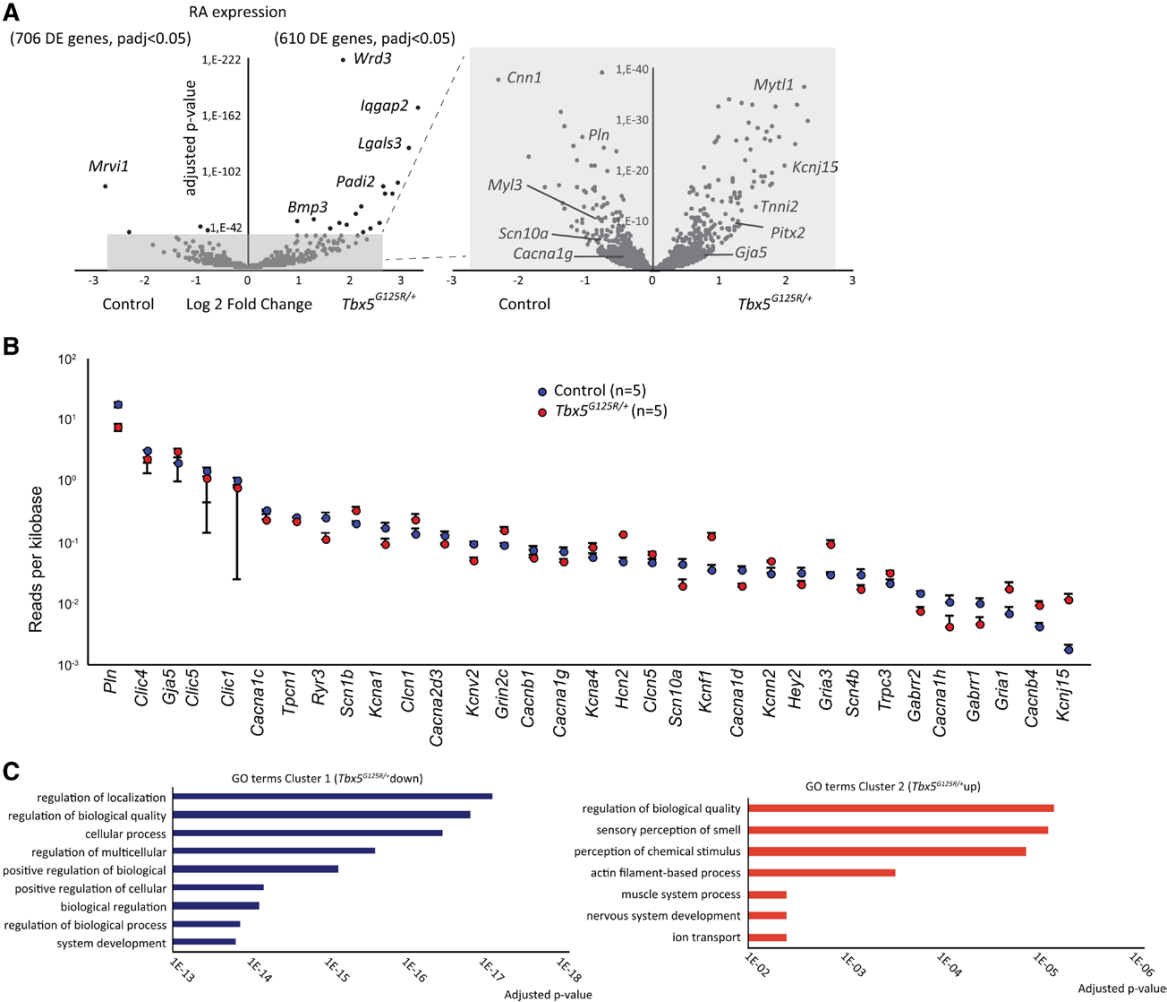
**Transcription analysis of whole tissue right atrium of adult Tbx5G125R/+ mice.** **A**, Expression analysis of the RA shows hundreds of genes differentially expressed between control (n=5) and *Tbx5*^*G125R/+*^ (n=5) atria. Differential expression analysis on whole-tissue RNA-sequencing was performed using the DESeq2 package. *P* values were corrected for multiple testing using the false discovery rate with 0.05 as control level. **B**, Expression analysis of ion handling genes between control and *Tbx5*^*G125R/+*^ mouse atria RNA-sequencing shows that 33 of 300 analyzed genes are differentially expressed (see Table S9; mean ± SEM, sorted by decreasing expression in the control group). **C**, GO-term analysis using PANTHER (protein analysis through evolutionary relationships). *Bmp3* indicates bone morphogenetic protein 3; Cacna1c, Calcium Voltage-Gated Channel Subunit Alpha1 C; Cacna1d, Calcium Voltage-Gated Channel Subunit Alpha1 D; Cacna1g, Calcium Voltage-Gated Channel Subunit Alpha1 G; Cacna1h, Calcium Voltage-Gated Channel Subunit Alpha1 H; Cacna2d3, Calcium Voltage-Gated Channel Auxiliary Subunit Alpha2delta 3; Cacnb1, Calcium Voltage-Gated Channel Auxiliary Subunit Beta 1; Cacnb4, Calcium Voltage-Gated Channel Auxiliary Subunit Beta 4; Clcn1, Chloride Voltage-Gated Channel 1; Clcn5, Chloride Voltage-Gated Channel 5; Clic1, Chloride Intracellular Channel 1; Clic4, Chloride Intracellular Channel 4; Clic5, Chloride Intracellular Channel 5; Ccn1, Cellular Communication Network Factor 1; DE, differentially expressed; Gabrr1, Gamma-Aminobutyric Acid Type A Receptor Subunit Rho1; Gabrr2, Gamma-Aminobutyric Acid Type A Receptor Subunit Rho2; Gja5, Gap Junction Protein Alpha 5; Gria1, Glutamate Ionotropic Receptor AMPA Type Subunit 1; Gria3, Glutamate Ionotropic Receptor AMPA Type Subunit 3; Grin2c, Glutamate Ionotropic Receptor NMDA Type Subunit 2C; GO, Gene Ontology; Hcn2, Hyperpolarization Activated Cyclic Nucleotide Gated Potassium And Sodium Channel 2; Hey2, Hes Related Family BHLH Transcription Factor With YRPW Motif 2; Iqgap2, IQ Motif Containing GTPase Activating Protein 2; Kcna1, Potassium Voltage-Gated Channel Subfamily A Member 1; Kcna4, Potassium Voltage-Gated Channel Subfamily A Member 4; Kcnf1, Potassium Voltage-Gated Channel Modifier Subfamily F Member 1; Kcnj15, Potassium Inwardly Rectifying Channel Subfamily J Member 15; Kcnn2, Potassium Calcium-Activated Channel Subfamily N Member 2; Kcnv2, Potassium Voltage-Gated Channel Modifier Subfamily V Member 2; Lgals3, Galectin 3; Myl3, Myosin Light Chain 3; Mytl1, Myelin Transcription Factor 1 Like; Padi2, Peptidyl Arginine Deiminase 2; Padj, P-adjusted value; Pln, Phospholamban; Pitx2, Paired Like Homeodomain 2; RA, right atrium; Ryr3, Ryanodine Receptor 3; Scn10a, Sodium Voltage-Gated Channel Alpha Subunit 10; Scn1b, Sodium Voltage-Gated Channel Beta Subunit 1; Scn4b, Sodium Voltage-Gated Channel Beta Subunit 4; Tbx5, T-box transcription factor 5; Tnni2, Troponin I2, Fast Skeletal Type; Tpcn1, Two Pore Segment Channel 1; and Trpc3, Transient Receptor Potential Cation Channel Subfamily C Member 3.

We found 1316 genes differentially expressed (*P*_adj_ < 0.05; Table S8; Figure 5A); 706 were downregulated in the *Tbx5*^*G125R/+*^ atria, 610 were upregulated. While the cardiac function of many of the most significantly differentially expressed genes (Table S8) has not been defined, *Lgals3* (galectin-3) and *Padi2* (peptidyl arginine deiminase-2) have been implicated in cardiac dysfunction.^17,18^ We observed differential expression in 33 of 300 analyzed gene-encoding proteins involved in ion handling/electrophysiology, among which were several intracellular Ca^2+^-modulating and gap junction genes (eg, *Cacna1c* [calcium voltage-gated channel subunit α1C], *Pln* [phospholamban], *Gja5* [gap junction protein α5], and *Slc8a1* [solute carrier family 8 member A1] which could account for the low Ca^2+^ transient amplitudes observed in *Tbx5*^*G125R/+*^ mice), as well as K^+^ channel genes such as *Kcnj15* (potassium inwardly rectifying channel subfamily J member 15) and *Kcnn2* ([potassium calcium-activated channel subfamily N member 2] Figure 5B; Table S9). Reduced expression of *Scn5a* (sodium voltage-gated channel α subunit 5), *Ryr2* (ryanodine receptor 2), and *Atp2a2* (ATPase sarcoplasmic/endoplasmic reticulum Ca^2+^ transporting 2) was linked to conduction slowing and altered intracellular Ca^2+^ concentrations in *Tbx5* haploinsufficient mice.^8–11^ However, these and other genes that were previously found to respond to Tbx5 insufficiency (*Nppa* [natriuretic peptide A] *Tbx3* [T-box transcription factor 3], *Dsp* [desmoplakin], *Gja1* [gap junction protein α1]^5, 9, 10^ [Table S8]) were not responsive to Tbx5-p.G125R. Gene Ontology term analysis yielded general terms for genes downregulated in the *Tbx5*^*G125R/+*^, but pointed to actin filament and muscle process, as well as ion transport, for upregulated genes (Figure 5C; Tables S10 and S11). Transcriptional profiles of left atria of mutants and controls were in accordance with those of the right atria (Table S8).

We explored fibrotic tissue markers (*Col1a1* [collagen type I α1 chain], *Col3a1* [collagen type III α1 chain], *Ctgf* [connective tissue growth factor], *Tgfb1* [transforming growth factor β1], *Postn* [periostin]) in whole-tissue RNA-seq of right and left atria, of which only *Postn* (activated fibroblast marker) was decreased in expression in right atria of *Tbx5*^*G125R/+*^ animals (Figure S6B; Table S8). These data further support the absence of fibrosis and activated fibroblasts in *Tbx5*^*G125R/+*^ atria. Furthermore, as the right atrial samples all contained sinoatrial node tissue, we explored the expression of sinoatrial node–specific genes^19–23^ in the whole-tissue RNA-seq data. Of 30 markers tested, 14 showed differential expression. Notably, transcripts for key TFs Tbx3, Isl1 (insulin gene enhancer protein ISL-1), and Shox2 (short stature homeobox 2), and key ion channels Hcn4 (hyperpolarization activated cyclic nucleotide gated potassium channel 4), Hcn1 (hyperpolarization activated cyclic nucleotide gated potassium channel 1), and Cacna2d2 (calcium voltage-gated channel auxiliary subunit α2 δ2) were not differentially expressed between genotypes. However, *Cacna1d*, (calcium voltage-gated channel subunit α1 D), *Cacna2d3*, calcium voltage-gated channel auxiliary subunit α2 δ3), *Cacna1g* (calcium voltage-gated channel subunit α1 G), *Cacna1h* (calcium voltage-gated channel subunit α1 H), and *Ryr3* (ryanodine receptor 3), as well bone morphogenetic protein (BMP)–signaling components (*Bmp2*, *Bmp3*, *Bmp10* [bone morphogenetic proteins 2, 3, and 10, respectively], *Bmpr1b* [bone morphogenetic protein receptor type 1B]), showed significant differential abundance in the data sets (*P*_adj_ < 0.05; Table S8).

### Tbx5-p.G125R Induces Changes in Epigenetic States in Atrial CMs

**Figure 6.**
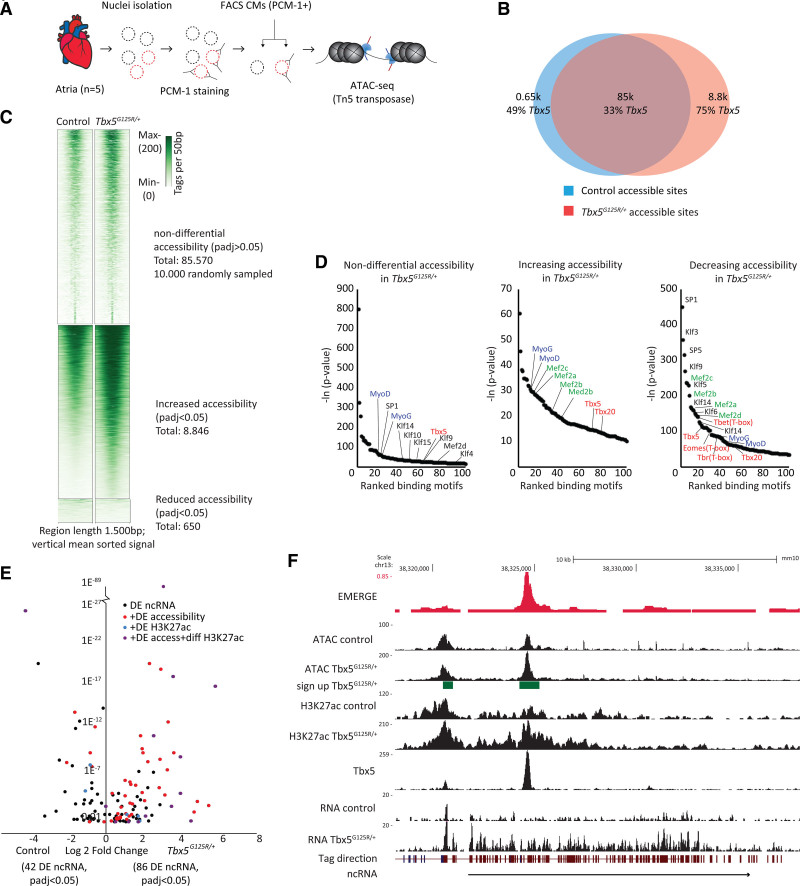
**ATAC-sequencing shows thousands of newly accessible chromatin overlapping Tbx5 binding sites in Tbx5G125R/+ mice.** **A**, Method of atrial CM nucleus isolation and ATAC-seq. **B**, Venn diagram showing overlap of accessibility sites in control and *Tbx5*^*G125R/+*^ mouse atria, as well as the overlap between accessibility sites and *Tbx5* binding sites (in %). **C**, Heatmap clustering showing increasing and decreasing accessibility. Differential accessibility was assessed using the DESeq2 package. *P* values were corrected for multiple testing using false discovery rate. **D**, HOMER motif analysis shows reduced accessibility sites in *Tbx5*^*G125R/+*^ atria were highly enriched for motifs of SP, MEF2, KLF, and TBX families of TFs, whereas Myo family member sites are enriched in increasingly accessible regions. False discovery rate adjusted *P* values are reported by HOMER. **E**, ncRNA expression analysis was performed using Limma to determine differential expression. **F**, University of California, Santa Cruz track showing a ncRNA upstream of *Bmp6* (bone morphogenetic protein 6) and differential accessibility. ATAC-seq indicates assay for transposase-accessible chromatin sequencing; CM, cardiomyocyte; DE, differential expression; diff, differential; Eomes (T-box), Eomesodermin; FACS, fluorescence-activated cell sorting; H3K27Ac, histone H3 lysine 27 acetylation; HOMER, Hypergeometric Optimization of Motif Enrichment; Klf10, Krüppel-like factor 10; Klf14, Krüppel-like factor 14; Klf16, Krüppel-like factor 16; Klf3, Krüppel-like factor 3; Klf4, Krüppel-like factor 4; Klf9, Krüppel-like factor 9; Mef2a, myocyte enhancer factor-2a; Mef2b, myocyte enhancer factor-2b; Mef2c, myocyte enhancer factor-2c; Mef2d, myocyte enhancer factor-2d; MyoD, Myogenic Differentiation; MyoG, Myogenin; ncRNA, noncoding RNA; *P*_adj_, *P*-adjusted value; PCM-1, pericentriolar material-1; SP1, specificity protein 1; SP5, specificity protein 5; Tbet (T-box), T-Box Transcription Factor 21; Tbr (T-box), T-Box Brain Transcription Factor 1; Tbx20, T-box transcription factor 20; TF, transcription factors; and Tn5, transposon 5.

To gain insight into the changes in epigenetic state and regulatory element deployment in response to Tbx5-p.G125R, we assessed the chromatin accessibility profile of control and *Tbx5*^*G125R/+*^ atrial CMs (n=4 each) using ATAC-seq (Figure 6A). There were 85 570 sites that showed similar accessibility between control and *Tbx5*^*G125R/+*^ atrial CMs, 8846 showed increased accessibility, and 650 showed decreased accessibility (Figure 6B and 6C). To identify Tbx5-occupied accessible sites, we crossed a chromatin immunoprecipitation followed by sequencing dataset of Tbx5 occupancy in the fetal mouse heart.^24^ There was a striking enrichment of Tbx5 occupancy, both in sites of gained (6634/8846; Z-test *P* < 0.001) and reduced accessibility (318/650; Z-test *P* < 0.0001) compared with sites of unchanging accessibility (26 664/85 570; Figure 6B). The sites of increasing as well as decreasing accessibility were enriched in motifs for MEF2 (myocyte enhancer factor-2) TF family members as revealed by HOMER (Hypergeometric Optimization of Motif Enrichment) analysis (Tables S12–S14; Figure 6D). Indeed, heart development involves physical interaction between TBX5 and MEF2C (myocyte enhancer factor 2C).^25^ The sites of decreasing accessibility were strikingly enriched for motifs for KLF/SP TFs (Figure 6D; Table S14). TBX5 was reported to interact and cooperatively regulate cardiac target gene expression with KLF13 (Krüppel-like factor 13).^26^ These data suggest that Tbx5-p.G125R may have reduced interaction with KLF/SP factors, leading to reduced accessibility of sites normally co-occupied by Tbx5 and KLF/SP factors.

To further examine the impact of Tbx5-p.G125R on genome-wide regulatory element activity, we examined the genomic localization of H3K27ac association by CUT&RUN in isolated PCM1+ (pericentriolar material 1) CM nuclei of 8-week-old control (n=5) and *Tbx5*^*G125R/+*^ (n=6) pooled left and right atria (Figure S7A). We detected 127 286 regions enriched for H3K27ac association and 1653 sites with significantly differential H3K27ac regions (*P* < 0.05; Table S15), of which 1335 regions have increased signal in *Tbx5*^*G125R/+*^ and 318 regions have reduced signal (Figure S7B). Of the 127 286 H3K27ac regions, 18% (22 911/127 286) overlapped with Tbx5 binding sites in the fetal mouse heart^24^ (Table S16), whereas in the regions with increased H3K27ac association, 40% (347/1335; Z-test *P* < 0.0001) overlapped Tbx5 binding sites (Figure S7C). For example, accessibility and H3K27ac signal of Tbx5-bound regulatory elements in *Padi2* and *Wdr3* (WD repeat-containing protein 3) are strongly increased in *Tbx5*^*G125R/+*^ atria (Figure S7E and S7F). Interestingly, the H3K27ac regions that are reduced in the *Tbx5*^*G125R/+*^ atria all overlap Tbx5 binding sites (318/318; Z-test *P* < 0.0001; Figure S7C). These data are in agreement with the chromatin accessibility analysis, and together indicate that Tbx5-p.G125R induces altered epigenetic states of sites (including putative enhancers) normally occupied by Tbx5 and—to a lesser extent—of sites not occupied by Tbx5.

Previously, Tbx5-dependent enhancers and noncoding RNA (ncRNA) were identified by analyzing Tbx5-dependent enhancer transcription.^12^ By crossing our RNA-seq data with the ATAC-seq and CUT&RUN data sets (see Supplemental Methods; Table S17), we detected 128 enhancer-derived transcripts (ncRNA, transcriptional start site colocalizing with accessible and H3K27ac-associated site) that were differentially expressed between control and *Tbx5*^*G125R/+*^ animals in the right atria (n=5; *P*_adj_ < 0.05), of which 42 were more abundant in the control and 86 more abundant in the *Tbx5*^*G125R/+*^ animals (Figure 6E; Table S17). Out of the 128 thus identified elements, 71 showed Tbx5 occupation (Table S17).^24^ The remaining 57 ncRNA-associated elements often showed Tbx5 occupation of accessible/H3K27ac-marked elements at a few-kbp distance, but these were considered to not colocalize. An example of a differentially expressed ncRNA with a transcriptional start site overlapping differential accessibility and H3K27ac is shown in Figure 6F near *Bmp6* (bone morphogenetic protein 6). Of these differentially active regulatory elements, we identified 166 candidate target genes that were differently expressed between genotypes (Table S17). Highly interesting candidates include *Tbx20* (T-box transcription factor 20), *Sema3a* (semaphorin 3A), *Kcna4* (potassium voltage-gated channel subfamily A member 4), *Slc8a1*, and *Tgfb2* (transforming growth factor β2), which are likely to impact atrial function in *Tbx5*^*G125R/+*^ animals (Table S17, column N)^27–31^ Differentially transcribed ncRNAs, in combination with differential accessibility and differential target gene expression, are shown for the *Tgfb2*, *Sema3a*, and *Kcnj15* loci in Figure S8. Interestingly, previously identified prominent Tbx5-dependent ncRNAs (close to *Ryr2* and to *Atp2a2* [Serca2a (sarcoplasmic/endoplasmic reticulum CA^2+^ ATPase 2a])^10,12^ and their putative target genes (*Ryr2*, *Atp2a2*) were not affected in *Tbx5*^*G125R/+*^ atria (Figure S9).

## Discussion

Previous characterization of a Dutch family with the pathogenic TBX5-p.G125R missense variant revealed atypical HOS, with mild skeletal abnormalities and lower penetrance of congenital heart defects but a high prevalence of AF.^15^ Here, we show that these patients suffer from other types of supraventricular arrhythmias that indicate dysfunctionality of the atrial muscle, including the sinoatrial node and AV node, that were not identified previously (Table S1). Typical HOS is usually associated with loss-of-function variants leading to haploinsufficiency; the atypical HOS phenotype of *TBX5-p.G125R* carriers suggest the pathogenic missense variant causes TBX5 to gain specific function(s). In the mouse model with this variant, the quantity of *Tbx5* (wild-type) and *Tbx5-p.G125R* mRNA is equal in the adult *Tbx5*^*G125R/+*^ mouse atria, indicating that loss of expression (ie, by nonsense-mediated decay) causing haploinsufficiency does not occur. We did identify broadly changed expression profiles and epigenetic states induced by heterozygosity for Tbx5-p.G125R. However, important genes and enhancers previously found to respond to Tbx5 insufficiency (eg, *Ryr2*, *Atp2a2*, *Nppa*, *RACER*)^5,9,10,12^ did not respond to Tbx5-p.G125R. This indicates Tbx5-p.G125R has gained functions invoking molecular mechanisms different from those invoked by reduced Tbx5 dose. Intriguingly, expression quantitative trait loci analysis revealed that AF risk variant carriers in the human population express significantly more, not less, *TBX5* in the heart.^14^ It will be interesting to investigate whether the Tbx5-p.G125R TF and increased TBX5 dose in risk variant carriers share transcriptional responses underlying AF propensity.

The patients displayed AV junctional escape beats, atrial extras, sinus bradycardia, and sick sinus syndrome in addition to AF. These arrhythmias were reproduced in the Tbx5-p.G125R mouse model, indicating Tbx5-p.G125R function and impact on cardiac electrophysiology in vivo are conserved between human and mice. Tbx5 is critical for the development and gene regulation in the sinoatrial and the AV nodes.^3,5,6,32,33^ The right atrial RNA-seq data sets indicated differential expression in 14 of 30 selected sinoatrial node markers. While these findings require validation, they suggest that the sinoatrial node may be transcriptionally affected in *Tbx5*^*G125R/+*^ mice. The differentially expressed genes included genes encoding calcium channels and BMP-signaling components important for sinoatrial node development and function,^20,22^ which may contribute to dysfunction of the sinoatrial node in *Tbx5*^*G125R/+*^ mice. Moreover, the balance between Tbx5 and the transcriptional repressor Tbx3 was found to determine the function of conduction system components.^34,35^ The altered DNA binding properties of Tbx5-p.G125R could affect both the regulation of important regulators in the nodes, and change the competitive balance with Tbx3. Further studies are required to explore the impact and underlying mechanisms of Tbx5-p.G125R on the sinoatrial and AV nodes.

Transcriptional profiling of the atria showed that heterozygosity of Tbx5-p.G125R leads to extensive dysregulation of gene expression in the atria, indicating multiple and complex mechanisms underlie the arrhythmia phenotype. While studies of *Tbx5*-haploinsufficient and -deficient mouse models implicate reduced expression of *Ryr2* and *Atp2a2* in AF propensity,^9,11,36^ these genes are not deregulated in atria of heterozygous Tbx5-p.G125R mice. However, we observed changes in expression of many other ion-handling protein-encoding genes, and in particular, genes associated with atrial function. Notably, the transcriptional profiling and differential regulatory element usage analysis identified Tbx5-p.G125R–mediated increased atrial expression of *Tbx20*, *Sema3a*, *Kcna4*, and *Pitx2c* (Paired Like Homeodomain 2 isoform c; the latter of which is specifically in the right atrium), and decreased atrial expression of *Tgfb2*. Each of these genes has been causally linked to heart function and rhythm control, further indicating that multiple complex mechanisms underlie the arrhythmia phenotype. Tbx20 is an essential cardiac TF, controlling CM function, ion channel gene expression, and atrial development.^27,28^ Because T-box TF function is highly dose-sensitive, increased Tbx20 expression in *Tbx5*^+/G125R^ atria is likely to broadly affect cardiac gene expression and function. Sema3a, produced by CMs, controls cardiac sympathetic innervation and is associated with arrhythmia when disrupted or overexpressed in mice.^29^ Moreover, Sema3a was identified as a naturally occurring Kv4.3 (potassium voltage-gated channel) inhibitor,^37^ suggesting its induction could affect Kv4.3. An induction of Kv1.4 (encoded by *Kcna4*) has been observed in pathophysiologic conditions in the human heart (see Ni et al^31^). An increase of Kv1.4 (partly replacing Kv4.3) in atria was suggested to prolong ADP and to alter its dynamics, causing AF predisposition.^31^

Pitx2c is specifically expressed in the left atrium, where it imposes left identity on the atrium, initiates pulmonary vein myocardium formation, and suppresses sinoatrial node development during embryogenesis.^38–40^ In the formed left atrium, Pitx2c was shown to interact with Tbx5 to regulate ion channel gene expression and electrophysiology.^10^ Ectopic induction of *Pitx2c* in the right atrium could potentially affect sinoatrial node development and function, consistent with the observation that both human and mouse TBX5-pG125R carriers display sinoatrial node dysfunction. Furthermore, the atrial TF network around Tbx5 is likely affected by ectopic presence of Pitx2c in the right atrium, leading to altered ion channel gene expression.^10^ The decreased expression of *Tgfb2* in the atria of heterozygous Tbx5-p.G125R mice may lead to reduced TGF-β signaling and diminished fibrosis,^30^ as is observed in the atria of these mice.

Patch clamp measurements of isolated atrial *Tbx5*^*G125R/+*^ CMs show a prolongation of the APD. While APD is often shortened in AF patients, mainly in the setting of sustained arrhythmias,^41,42^ APD prolongation is associated with increased risk of developing AF in patients.^43–45^ In atria of *Tbx5*^*G125R/+*^ mice, APD was prolonged as well, which is consistent with the patient clinical picture as well as with the increase in atrial arrhythmias we observed. The decreased Ca^2+^ amplitude and intracellular Ca^2+^ concentrations may be a result of longer Ca^2+^ currents because of reduced Ca^2+^-induced Ca^2+^ current inactivation, as well as decreased activation of Ca^2+^-activated K^+^ channels, and both mechanisms may contribute to the prolonged ADP that was observed. Moreover, deregulation of genes encoding channel subunits for various voltage-gated potassium currents (eg, *Kcna1* [Kv1.1], *Kcnj15* [Kir4.2 (inwardly rectifying potassium channel 4.2)], *Kcna4* [Kv1.4]; see Figure 5B) may also contribute to prolonged ADP. Decreased expression of genes encoding intracellular Ca^2+^-modulating proteins such as *Cacna1c* and *Cacna1g* could be at the basis of the decreased of Ca^2+^ transient amplitude observed in the mutant mice. Another explanation could involve *Mrvi1* (murine retrovirus integration site 1 homolog), which uniquely responds to Tbx5-p.G125R and is decreased in the *Tbx5*^*G125R/+*^ atria. Mrvi1 is associated with Pln and plays a key role in cardiac contractility by modulating nitric oxide–induced inhibition of Ca^2+^ signaling.^46,47^

A relatively large number of sites with increased accessibility and with increased H3K27ac association were observed in heterozygous Tbx5-p.G125R atrial CMs. These sites were enriched for Tbx5 binding in the normal heart,^24^ suggesting TBX5-p.G125R may display increased interaction with genuine Tbx5-occupied sites in vivo, in keeping with its increased DNA-binding affinity in vitro.^15^ However, sites normally not occupied by Tbx5 also showed increased accessibility, and the sites displaying decreased accessibility were enriched for Tbx5-binding and T-box motifs. Together, these observations suggest Tbx5-p.G125R has acquired properties that both quantitatively and qualitatively influence chromatin interaction. Additional analyses (eg, chromatin immunoprecipitation followed by sequencing, proteomics) are required to define these altered interaction properties of Tbx5-p.G125R. The changed chromatin interaction may involve a shift in affinity for binding partner TFs or cofactors, as was previously also reported for missense mutations in GATA4 (GATA-binding protein 4) and NKX2-5.^48,49^ The sites of reduced accessibility in heterozygous mutants were highly enriched for motifs of Sp/KLF, MEF2, and T-box families of TFs, pointing to candidate TFs that normally interact with Tbx5, and to a lesser extent, Tbx5-p.G125R. Mef2c and Klf13 are reported functional interaction partners of Tbx5 in the regulation of cardiac development and gene regulation.^25,26^ The Sp4 TF is expressed in the heart, including the atria and AV node, and Sp4-deficient mice display a high incidence of sinus bradycardia and AV block.^50^ Future studies could explore whether the Tbx5-p.G125R interactions with Mef2c—as well as the Sp and Klf TF family members expressed in the atria—are affected.

In conclusion, Tbx5-p.G125R displays altered properties that induce changes in the epigenetic state, such as chromatin accessibility and H3K27ac, that underlie deregulated expression of both Tbx5 target genes and off-target genes, which is at the basis of distinct electrophysiologic changes that recapitulate clinical observations in patients heterozygous for TBX5-p.G125R.

## Article Information

### Acknowledgments

We thank Berend de Jonge, Aho Ilgun, and Corrie de Gier-de Vries for their contributions. We thank Jan Ruijter for assisting with statistical analysis and presentations.

### Sources of Funding

This work was supported by CardioVasculair Onderzoek Nederland (RACE-V Acceleration and Career Development to A.F.O.; project 2014-18 CONCOR-genes Young Talent Program to F.M.B.; and project 2014-18 CONCOR-genes to V.M.C.), Leducq Foundation (14CVD01 to V.M.C.), and Dutch CardioVascular Alliance (OUTREACH to V.M.C.).

### Disclosures

None.

### Supplemental Material

Supplemental Methods

Figures S1–S9

Table S1–S18

References 51–72

## Supplementary Material


