# Supplementary material for: Patient-Specific TBX5-G125R Variant Induces Profound Transcriptional Deregulation and Atrial Dysfunction
Source: Circulation. 2022 Feb 3;145(8):606–19. doi: 10.1161/CIRCULATIONAHA.121.054347 (PMC8860223; doi:10.1161/CIRCULATIONAHA.121.054347)
Supplement: Supplementary file 1 [file cir-145-606-s001.pdf]

van Ouwerkerk et al. Patient-specific TBX5-G125R variant induces profound transcriptional deregulation and atrial dysfunction.

SUPPLEMENTAL MATERIAL

Supplemental Methods

Statistics. For statistical analysis SPSS was used unless otherwise indicated. Because of the non-normal distribution of some data sets (Shapiro-Wilk test) all observed differences between the two genotypes were tested for statistical significance with the Mann-Whitney U test. When the two unpaired groups were embedded in a two or three way experimental design, an ANOVA test was performed to determine whether the genotype effect was dependent on the level of the other factors in the design; such dependence was defined as a significant interaction term in ANOVA. Differences between fractions were tested with a Z-test as implemented in SAGEstats.[49] A p-value less than 0.05 was considered statistically significant.

Specific analysis packages were employed for the analysis of large sets of sequencing date. In these cases, between group differences were tested, and corrected for multiple testing, with the methods available in those packages. Specifically, differential gene expression in snRNA-seq was determined using the R2 platform (R2: Genomics Analysis and Visualization Platform (http://r2.amc.nl)) which uses the Kruskal-wallis test and Bonferroni multiple testing correction. Differential expression analysis on whole tissue RNA-seq, differential accessibility as well as differential acetylation were compared using the DESeq2 package based on a model using the negative binomial distribution with false discovery rate (FDR) correction for multiple testing.[50] To determine whether the G125R variant was more often, compared to the wildtype allele, present in the RNA-seq reads, the Fisher's exact test was performed on the observed allele frequencies. GO-term analysis on snRNA-seq differentially expressed genes was performed using PANTHER Version 16.0,[51] with Fisher overrepresentation test and FDR correction. The same package, with Bonferroni correction, was used for GO-term analysis on whole tissue RNA-seq differentially expressed genes. Limma was used to determine ncRNA differential expression.[52] Motif discovery was performed with HOMER and FDR correction).[53]

Genome editing. 25 ng/ul Cas9 mRNA (PmeI linearized MLM3613 vector transcribed using mMessage mMachine T7 Ultra kit and purified using Megaclear kit), as well as 10 ng/ul sgRNA (Supplemental Table 18) (DraI linearized DR274 vector Megashortscript kit T7 + Megaclear kit) and 10 ng/ul 200nt PAGE Ultramer DNA Oligo (PAGE purified; IDT DNA) was injected into the pro-nucleus of an FVB zygote. The ssODN of 200bp (Supplemental Table 18), contained the p.G125R mutation in the middle. Genotyping of each mouse was performed on DNA extraction by alkaline lysis method, and PCR using hotfire polymerase (Solis BioDyne) (95°C 3 minutes, (35x 95°C 30 seconds, 64°C 30 seconds, 72°C 1 minute) final elongation 72°C 5 minutes) using forward and reverse primers (Supplemental Table 18), 1ul of ExoSAP-IT PCR Product Cleanup Reagent (ThermoFisher) was added to 2,5ul of PCR reaction for

cleanup of PCR product (37°C 4 minutes, 80°C 1 minute), followed by BigDye Terminator v3.1 (ThermoFisher) Sanger sequencing using the reverse primer only.

Tissue harvest. Adult mice were asphyxiated using CO_2 followed by cervical dislocation. Pregnant mice used for embryo collection were anaesthetized using 5% isoflurane for 5 minutes followed by cervical dislocation and embryo harvest was performed in 1x PBS at 4°C.

EdU cardiomyocyte proliferation

Timed pregnant female mice received intraperitoneal injected of EdU sixty minutes before sacrifice by isoflurane and cervical dislocation. E12.5 Embryos were isolated in 1xPBS on ice, the head was removed and the body fixated in 4% PFA for 24 hours. On wildtype (n=5), *Tbx5*$^{+/G125R}$ (n=4) and *Tbx5*$^{G125R/G125R}$ (n=5) embryos, 7 µm sections were stained with mouse-anti-actin (1:400; Sigma), goat-anti-nkx2.5 (1:150; Santa Cruz), DAPI (1:1000) and Click-IT (ThermoFisher) before imaging and counting. Nkx2.5 positive nuclei were designated cardiomyocytes, Nkx2.5 and EdU double positive nuclei were designated proliferating cardiomyocytes. Ratios were normalized within different litters, Shapiro-Wilk test was used to test for normal distribution, and ANOVA was performed on the normalized ratios.

ECGs and Trans-esophageal burst pacing. All procedures as described elsewhere. In this experiment male (control n=22, HET n=10) as well as female mice (control n=12, HET n=8) were used of ages between 12-16 weeks, and the scientist was blinded for genotype during ECG measurements, pacing and data analysis. In short, mice were anaesthetized using 5% isoflurane, and after disappearance of reflexes the animals were placed on a heating pad of 37°C with a steady flow of isoflurane 1.5%. Electrodes were inserted subcutaneously in the limbs and connected to an electrocardiogram (ECG) amplifier (PowerLab 26T, ADInstruments, Oxford, United Kingdom). ECG was measured for 5 min before further pacing experiments. Electrophysiology studies were performed as described before. [54, 55] In short: For atrial stimulation, an octapolar catheter was advanced through the esophagus to the site where capture of the atria could be established. The transesophageal stimulation electrodes were connected to an isolation unit through which triggered stimuli with variable current output were initiated. For sinus node recovery time (SNRT) measurements, a 4 s pacing train with a cycle length of 100 ms was applied. SNRT definition: interval between the last pacing stimulus and onset of first sinus return. To control for differences in sinus rate, SNRT was normalized to resting heart rate (cSNRT = SNRT – RR interval) before pacing. Wenckebach period was determined by incremental increases in atrial pacing frequency performed for 2 s, beginning at 100 ms. Atrial and atrioventricular (AV) nodal effective refractory periods were determined by 1-s stimulus trains at 100 ms cycle length with a

shorter last stimulus cycle of 90 ms decreasing successively by 2 ms (s1-s2 loop) until AV block was achieved. Atrial arrhythmia inducibility was determined by 1 or 2 s bursts with cycle length of 60 ms, decreasing successively with a 2 ms decrement – measured down to a cycle length of 10 ms. AA definition: period of rapid irregular atrial rhythm lasting at least 1 s.

Optical mapping. The mice (8 control and 8 $Tbx5^{+/G125R}$) were killed by cervical dislocation after which the hearts were excised and the aorta cannulated. The hearts were Langendorff-perfused with Tyrode's solution (37°C) containing: NaCl 128 mM, KCl 4.7 mM, CaCl2 1.45 mM, MgCl2 0.6 mM, NaHCO3 27 mM, NaH2PO4 0.4 mM, and glucose 11 mM. The solution was maintained at 7.4 pH by equilibration with a mixture of 95% O_2 and 5% CO_2 and contained 10 µM Blebbistatin (Bio-Techne LtD) to reduce motion artefacts in the optical signals. The hearts were incubated – not perfused – in 10 ml Tyrode's containing 15 µM Di-4 ANEPPS (Molecular Probes, Eugene, OR) after which the hearts were placed in the optical mapping setup and submerged in HEPES buffered Tyrode's solution. Pseudo-electrograms were recorded by placing 3 electrodes at ±0.5 cm distance of the heart in the Einthoven configuration. Electrode R and L were placed alongside the right and left atrium, respectively, whereas electrode F was placed alongside the apex. Electrode R was used as negative input for both lead I and lead II. Recordings were made using Labchart amplifier (AD Instruments, Model 15T, sample frequency 1 kHz) and analyzed in LabChart Pro v8.1.13. Excitation light was provided by a 5 Watt power LED (filtered 510 ± 35 nm). Fluorescence (filtered > 610nm) was transmitted through a lens system on CMOS sensor (100 x 100 elements, sampling rate 5 kHz, MICAM Ultima). Optical action potentials were analyzed with custom made software. Atrial conduction velocity was determined during central stimulation using a custom made current controlled stimulator (Antronics).

Patch clamp. Mouse hearts were harvested from male mice of 8 to 16 weeks of age (n=7 wildtype and n=4 $Tbx5^{+/G125R}$). Single cells were isolated from left atria by enzymatic dissociation. Therefore, excised hearts were perfused for 5 minutes in a Langendorff system with a modified Tyrode's solution containing (in mmol/L): NaCl 140, KCl 5.4, $CaCl_2$ 1.8, $MgCl_2$ 1.0, glucose 5.5, HEPES 5.0; pH 7.4 (set with NaOH). Subsequently, the hearts were perfused with Tyrode's solution containing a low Ca^{2+}-concentration (10 µmol/L) for 10 minutes, after which Liberase TM research grade (Roche Diagnostics, GmbH, Mannheim, Germany) and Elastase from porcine pancreas (Bio-Connect B.V., Huissen, Netherlands) were added for 12 minutes at a concentration of 0.038 mg/mL and 0.01 mg/mL, respectively. All solutions were saturated with 100% O_2 and the temperature was maintained at 37 °C. To obtain single cells, the digested left atria was cut into small pieces which were triturated for 4 minutes through a pipette (tip diameter: 0.8 mm) in the low Ca^{2+} Tyrode's solution, supplemented with 10 mg/ml Bovine Serum Albumin (Roche Diagnostics, essential fatty free, fraction V). Single cells were

stored at room temperature for at least 45 min before they were used. Quiescent single rod-shaped cells with smooth surfaces were selected for electrophysiological measurements.
Action potentials (APs) were recorded at 36±0.2 °C using the amphotericin-perforated patch-clamp technique with an Axopatch 200B amplifier (Molecular Devices, Sunnyvale, CA, USA). Data acquisition and analysis were performed with custom-made software. Patch pipettes (resistance 2–3 MΩ) were pulled from borosilicate glass capillaries (Harvard Apparatus, UK) using a custom-made microelectrode puller. Action potentials were low-pass-filtered with a cut-off of 5 kHz, and digitized at 40 kHz. Potentials were corrected for the calculated liquid junction.[56]

<u>AP measurements.</u> AP were measured using the modified Tyrode's solution as bath solution.. Pipettes were filled with solution containing (in mmol/l): K-gluc 125, KCl 20, NaCl 5, amphotericin-B 0.44, HEPES 10, pH 7.2 (KOH). APs were elicited at 2 to 8 Hz by 3-ms, ~1.2× threshold current pulses through the patch pipette. We analyzed resting membrane potential (RMP), maximal AP amplitude (APA), maximum AP upstroke velocity, and AP duration at 20, 50 and 90% repolarization (APD_{20}, APD_{50} and APD_{90}, respectively). Parameters from 10 consecutive APs were averaged.

<u>Ca^{2+} measurements.</u> CMs were isolated as described above. Intracellular calcium concentrations ($[Ca^{2+}]_i$) were measured at 37 degrees in HEPES solution ((mmol/l): $[Na^+]$ 156, $[K^+]$ 4.7, $[Ca^{2+}]$ 1.3, $[Mg^{2+}]$ 2.0, $[Cl^-]$ 150.6, $[HCO_3]$ 4.3, $[HPO^{2-}]$ 1.4, [Hepes] 17, [Glucose] 11 and 1% fatty acid free albumin, pH 7.3) using the fluorescent probe Indo-1 as described previously.[57] In brief, myocytes were exposed to the acetoxymethyl esters of either 5 μmol/l indo-1 during 30 min at 37°C. They were washed twice with fresh extracellular solution (without albumin) and kept for 15 min to ensure complete de-esterification. Myocytes were attached to a poly-D-lysine (0.1 g/l) treated cover slip placed on a temperature controlled (37°C) microscope stage of an inverted fluorescence microscope (Nikon Diaphot) with quartz optics. A temperature controlled perfusion chamber (height 0.4 mm, diameter 10 mm, volume 30 μL, temperature 37°C), with two needles at opposite sides for perfusion purposes, was tightly positioned over the cover slip. The contents of the chamber could be replaced within 100 ms. Bipolar square pulses for field stimulation (40 V/cm) were applied through two thin parallel platinum electrodes at a distance of 8 mm. One quiescent single myocyte was selected (myocyte with more than one spontaneous oscillation per 10 s were excluded) and the measuring area was adjusted to the rod-shaped surface with a rectangular diaphragm. The wavelength of excitation of Indo-1 was 340 nm, applied with a stabilized xenon-arc lamp (100 W) with flashes of 100 at duration at a frequency of 2 Hz. Fluorescence was measured in dual emission mode at 410 and 516 nm. Emitted light passed a barrier filter of 400 nm, a dichroic mirror (450 nm) and respective narrow band interference filters in front of two

photomultipliers (Hamamatsu R-2949). The sample rate of signals recorded during each flash was 10 kHz and averaged. Averaged signals were corrected for background as well as for separately measured signals from non-loaded myocytes. Apparent $[Ca^{2+}]_i$ was calculated according to the ratio equation.[58]

RNA isolation. RNA isolation of tissue for both qPCR and RNA-seq was done with ReliaPrep RNA Tissue Miniprep System (Promega), using the kit DNase step. RNA quantity and quality was tested using respectively Qubit RNA HS Assay (ThermoFisher) and Bioanalyzer High Sensitivity RNA analysis chip (Agilent).

RNA-seq. Truseq RNA library Prep Kit v2 (Part# 15031048 Rev.E, Illumina) was performed on adult RA and LA separately (n=5) using 300ng input, 10 cycli amplification and 2 rounds of AmPure cleanup (1.0x, Beckman Coulter). 10 samples were run on Hiseq 2000 SE50 (Illumina).

Nuclei isolation. Nuclei were isolated as described before.[59] All steps were performed at 4 °C. In short, tissue was cut into pieces <1mm on ice using razor blades. The tissue pieces were turraxed for 45 s at highest setting using Turrax in 2 mL Lysis buffer (10 mM Tris-HCl pH 8, 5 mM $CaCl_2$, 2 mM EDTA, 0.5 mM EGTA, 1 mM DTT, 3 mM MgAc) containing RNAse inhibitor (NEB) in a round bottom stoppertube. Nuclei suspension is then diluted with 3 mL Lysis buffer supplemented with 0.4% Triton-X, and transferred to a Douncer. Next, 10 strokes were performed with a loose pestle, 10 with a tight pestle. The lysate is passed through a 100 µm strainer (Sysmex), filter washed with 2 mL Lysis buffer supplemented with 0.2% Triton-X, passed through a 30 µm strainer (Sysmex), and the filter washed again with 1 mL Lysis buffer supplemented with 0.2% Triton-X. The resulting lysate is centrifuged for 5 minutes at 1000xg, the supernatant is poured off carefully, and the pellet of nuclei resuspended in Staining buffer (1x PBS with 1% BSA supplemented with RNAse inhibitor).

Droplet-based Sn-RNA-seq. Female mice of 9 to 14 weeks of age were used. Nuclei isolation was performed as described above on 3 right atria of each genotype pooled together loaded (resulting in one wildtype sample (containing n=3 RA) and one $Tbx5^{+/G125R}$ sample (containing n=3 RA)). Then nuclei were FACS sorted using a gate for DAPI into 0.4% BSA-PBS solution. Nuclei were counted manually using cell counter and 7000 nuclei were loaded. As per 10x Genomics protocol, sorted single nuclei were processed through the GemCode Single Cell Platform using GemCode Gel Bead, Chip, and Library Kits (10x Genomics). The nuclei were then partitioned into gel beads in emulsion in the GemCode instrument followed by processing. Libraries were sequenced on an Illumina Hiseq 4000 PE150. We filtered for at least 1000 reads per nucleus using R2 (R2; Genomics analysis and visualization platform (http://r2.amc.nl)). t-Distributed Stochastic Neighbor Embedding (*t*-SNE) is a dimensionality reduction

algorithm that reduces high dimensional datasets to 2 or 3 dimensions. Differential gene expression in snRNA-seq was determined by Kruskal-wallis test and Bonferroni correction in R2.

Cleavage under targets and release under nuclease (CUT&RUN).[60] Nuclei were isolated as described above from 8 week old wildtype (n=5) and *Tbx5*$^{+/G125R}$ (n=6) pooled left and right atrium. 100ul was taken from the isolated nuclei as a negative control for FACS gating. Nuclei were incubated with rabbit PCM-1-antibody (AtlasAB) (1:1000) for 30 minutes rotating at 4 C. Secondary antibody Donkey-anti-rabbit Alexa 647 (ThermoFisher) (1:500) was added and incubated for a further 30 minutes rotating at 4 C. Then, the sample was centrifuged at 1000xg for 5 minutes and resuspended in 500 µl staining buffer. DAPI was added (1:1000) and the sample was FACS sorted for PCM-1+ nuclei. 3000-5000 nuclei or 6000-30000 nuclei were sorted. Samples were collected in 300 µl staining buffer, centrifuged at 1000xg for 5 minutes, and resuspended in 600 µl NE buffer as stated in step 7 of the C&R procedure.[61] Protocol was followed as published, with 1:00 H3K27Ac incubation overnight. NEBNext Ultra II DNA library Prep Kit for Illumina was used with 15 cycli PCR. Fragment size was determined on a Tapestation high sensitivety chip, DNA concentration was determined with Qubit, and samples were pooled equimolilarly and sequenced PE150 on Hi-seq4000.

Chromatin accessibility analysis using ATAC-seq. Nucleus isolation was performed on control (n=4) and *Tbx5*$^{G125R/+}$ (n=4) atria as described above. We used two male and two female mice of 8-12 weeks for each group. ATAC-seq was performed on FACS-sorted PCM-1 positive nuclei (as described under CUT&RUN) using Illumina Tagment DNA enzyme and buffer (20034197) according to protocol. Libraries were sequenced paired end (PE150) on an Illumina Hiseq4000.

Differential expression analysis (RNA-seq). Reads were mapped to mm10 build of the mouse transcriptome using STAR. Differential expression analysis was performed using the DESeq2 package based on a model using the negative binomial distribution.[50] P-values were corrected for multiple testing using the false discovery rate (FDR) method of Benjamini-Hochberg. We have used 0.05 as FDR control level.

Peak calling and differential acetylation analysis (CUT&RUN). Reads from H3K27ac CUT&RUN data were mapped to mm10 build of the mouse genome using BWA,[62] the default settings were used. The BEDTools suite was used to distribute the genome wide signal into bins of 500 bp.[63] The background read level was determined per sample per 500 bp by taking the average read count in bins with less than 20 tags. The background level of tags was subtracted per bin per sample. Bins with less than 101 cumulative tags across all 11 samples were discarded as noise. Differential acetylation was assessed

using the DESeq2 package[50] based on a model using the negative binomial distribution P-values were corrected for multiple testing using the FDR. We have used 0.05 as FDR control level. Continuous bins with differential signal were subsequently merged together using the BEDTools suite.

Expression of variant alleles. STAR was used to map reads to mm10 build of the mouse transcriptome.[64] We used VarScan 2 to detect variants and their allele specific occurrence within the RNA-seq.[65] We used a minimum read depth of 100 and a minimum variant allele frequency threshold of 0.20 in VarScan 2 for variant detection. To determine whether the G125R variant was more often present in the RNA-seq reads compared to the wildtype allele, we performed Fisher's exact test on the observed allele frequencies.

ncRNA analysis. The RA RNA-seq dataset was used to calculate de novo transcripts using StringTie,[66] with a minimal transcript length 50bp. To distill the ncRNA from all detected transcripts, genes encoding for proteins (gencode.vM3.annotation on mouse genome build mm10 obtained from Biomart) subtracted from the StringTie result.
The Limma package was used to determine differential expression with settings from referenced paper, as it has shown superior performance in detecting ncRNAs.[67] P-values were corrected for multiple testing using FDR. We have used 0.05 as FDR control level. The resulting list of 159 differentially expressed ncRNAs contains a mix of MGI, RIKEN and not previously annotated transcripts; not all of them will be eRNAs. We considered a differential eRNA detection valid when a clear TSS could be determined (given the signature distribution of forward and/or reverse reads) and when we could establish colocalization with enhancer signatures at the TSS using EMERGE heart enhancer prediction[68] and the CUT&RUN and ATAC-seq data described in this manuscript. We detected 128 eRNAs to be differentially expressed in *Tbx5*$^{G125R/+}$.
To determine which ncRNAs are potentially relevant for nearby gene expression, promoter coordinates of genes within 2Mb of the differentially expressed ncRNAs were obtained by intersecting these coordinates using the BEDTools suite in Galaxy,[63] and expression results of these genes were extracted from the RNA-seq in this study. Additionally, myoblast TAD coordinates were obtained[69] to determine whether eRNA and potential (differentially expressed) target gene were within the same TAD (using BEDTools suite in Galaxy) (Supplemental Table 17).

Chromatin accessibility analysis using ATAC-seq. CM nucleus isolation was performed on control (n=4) and *Tbx5*$^{G125R/+}$ (n=4) atria as described above. ATAC-seq was performed on FACS-sorted PCM-1 positive nuclei using Illumina Tagment DNA enzyme and buffer (20034197) according to protocol. Libraries were sequenced paired end on an Illumina Hiseq4000.

Peak calling and differential accessibility analysis (ATAC-seq). Reads from ATAC-seq data were mapped to mm10 build of the mouse genome using BWA,[62] the default settings were used. The BEDTools suite was used to distribute the genome wide signal into bins of 500 bp.[63] Bins with less than 89 cumulative tags across all 10 samples were discarded as noise. Differential accessibility was assessed using the DESeq2 package based on a model using the negative binomial distribution.[50] P-values were corrected for multiple testing using FDR. We have used 0.05 as FDR control level. Continuous bins with differential signal were subsequently merged together using the BEDTools suite.

TF motif analysis using HOMER. 200bp summits were determined of ATAC-seq performed in mouse SAN cells.[70] These regions were intersected with H3K27Ac CUT&RUN peaks. In total, HOMER[53] was performed on sequences of 1,714 randomly sampled neutral peaks, 1,393 peaks up in Tbx5$^{+/G125R}$, 324 peaks down in Tbx5$^{+/G125R}$, all with random genome as background in HOMER.

Heatmap clustering using Pheatmap. Unsupervised hierarchical clustering was performed on differentially detected peaks and genes in RNA-seq, CUT&RUN and ATAC-seq using the R package pheatmap, version 1.0.8. (http://cran.r-project.org/web/packages/pheatmap/index.html).

Nkx2.5 immunostaining and cardiomyocyte counting. Mouse hearts were harvested from female mice of 30 to 36 weeks of age (n=4 wildtype and n=6 *Tbx5*$^{+/G125R}$), and fixed in 4% PFA for 24 hours. After an EtOH 70% wash, the hearts were embedded in paraffin and sectioned (5um). 1:10 sections with visible atrial tissue were used from immunohistochemistry of Nkx2.5. Sections were also stained for DAPI. Images were taken of 5-15 sections throughout the atria on a DSM5000 with 2.5 objective. In ImageJ, the Nkx2.5 and DAPI positive cells were quantified using automated counting of thresholded (threshold 200) particles. By dividing the number of nkx2.5 positive cells by the number of DAPI positive cells in the left atrium, we calculate the ratio of CMs. The average ratios of CMs per individual were compared using an unpaired t-test.

Fibrosis measurements (picrosirius red stainings). The same processed mouse hearts (n=4 wildtype and n=5 *Tbx5*$^{+/G125R}$) were used as described in the previous section ("cardiomyocyte counting"). 1:50 sections with visible atrial tissue were stained with picrosirius red. Stained sections were imaged (on a DSM5000 with 2.5x objective) with white balancing, corresponding to an image every 250um. Using ImageJ, (protocol "Quantifying Stained Liver Tissue" https://imagej.nih.gov/ij/docs/examples/stained-sections/index.html) the image is converted to grayscale, and all of the non-atrial tissue (and blood) is blacked out of the picture. The red-stained collagen isolated using thresholding, and measured (for

whole tissue measurement threshold 175, for fibrosis 110). Ratios are calculated by dividing percentage area of fibrosis by percentage area of whole tissue. On average 10 images were analyzed per atrium, of which the average was taken per individual.

Legends Supplemental Tables

Supplemental Table 1. Pedigree showing updated information from Postma et al., Circulation Research 2008, with additional electrical anomalies from re-analyzed ECG data.

Supplemental Table 2. Embryonic lethality of $Tbx5^{G125R/G125R}$ mice.

Supplemental Table 3. Heart size as a function of heart weight divided by tibia length of neonatal day 20 littermates.

Supplemental Table 4. Conduction velocity in isolated right atria.

Supplemental Table 5. Average calcium measurements of isolated atrial cardiomyocytes.

Supplemental Table 6. Single nucleus RNA-seq differential expression in different clusters. Differential expression determined by Kruskal-Wallis test and Bonferroni correction in R2.

Supplemental Table 7. GO term analysis of snRNA-seq differentially expressed genes in cardiomyocyte cluster.

Supplemental Table 8. Whole tissue RNA-seq of the right and left atrium.

Supplemental Table 9. Gene expression of 300 ion channel genes extracted from whole tissue RNA-seq. The 33 significantly different genes are depicted in Figure 5.

Supplemental Table 10. GO-term analysis using PANTHER of upregulated genes in whole-tissue RNA-seq of the right atrium.

Supplemental Table 11. GO-term analysis using PANTHER of downregulated genes in whole-tissue RNA-seq of the right atrium.

Supplemental Table 12. HOMER motif recognition in equally accessible sites in Tbx5 G125R heterozygotes and control atrial cardiomyocytes.

Supplemental Table 13. HOMER motif recognition in increased accessible sites in Tbx5 G125R heterozygotes compared to control atrial cardiomyocytes.

Supplemental Table 14. HOMER motif recognition in decreased accessible sites in Tbx5 G125R heterozygotes compared to control atrial cardiomyocytes.

Supplemental Table 15. Differential H3K27ac signals and overlap with promoters.

Supplemental Table 16. H3K27ac association (C&R) versus Tbx5 binding (ChIP-seq Akerberg 2019).

Supplemental Table 17. ncRNA expression data. Curated list of ncRNAs differential expression, overlap with epigenetic data (ATAC-seq, H3K27ac and Tbx5 occupancy) and presence of differentially expressed genes in a 1Mb and within the same TAD.

Supplemental Table 18. Oligonucleotides.

Supplemental Figure 1

A

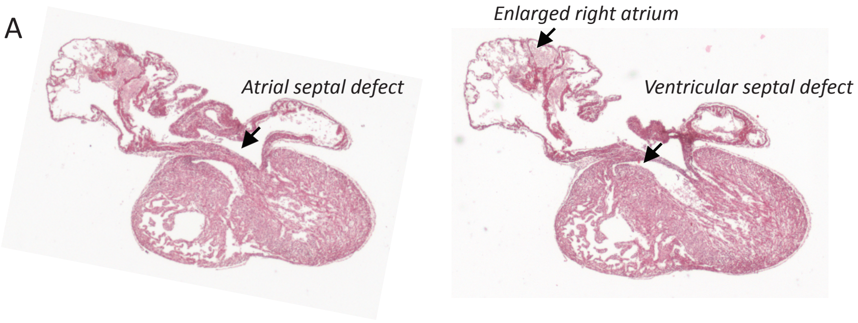

B

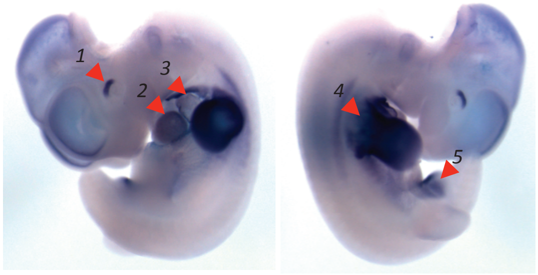

Control E10.5 embryo whole mount in situ of Tbx5

C

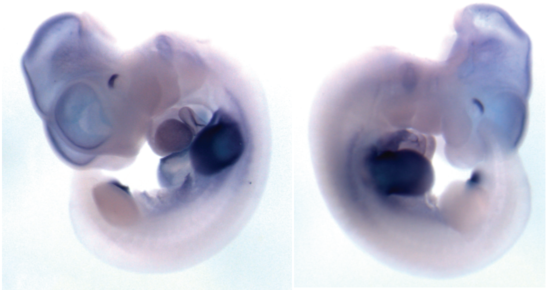

$Tbx5^{G125R/+}$ E10.5 embryo whole mount in situ of Tbx5

Supplemental Figure 2

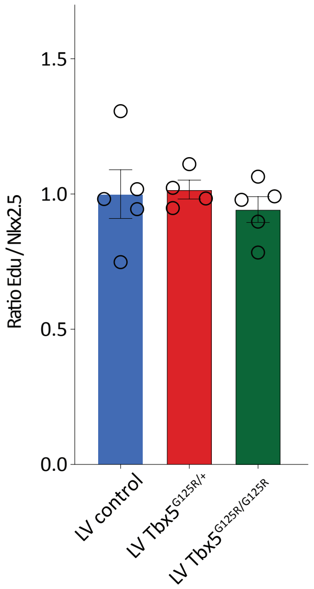

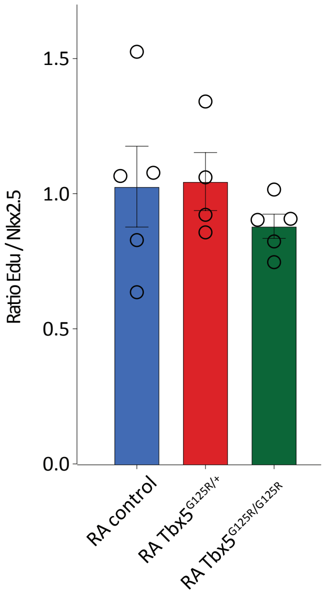

Supplemental Figure 3

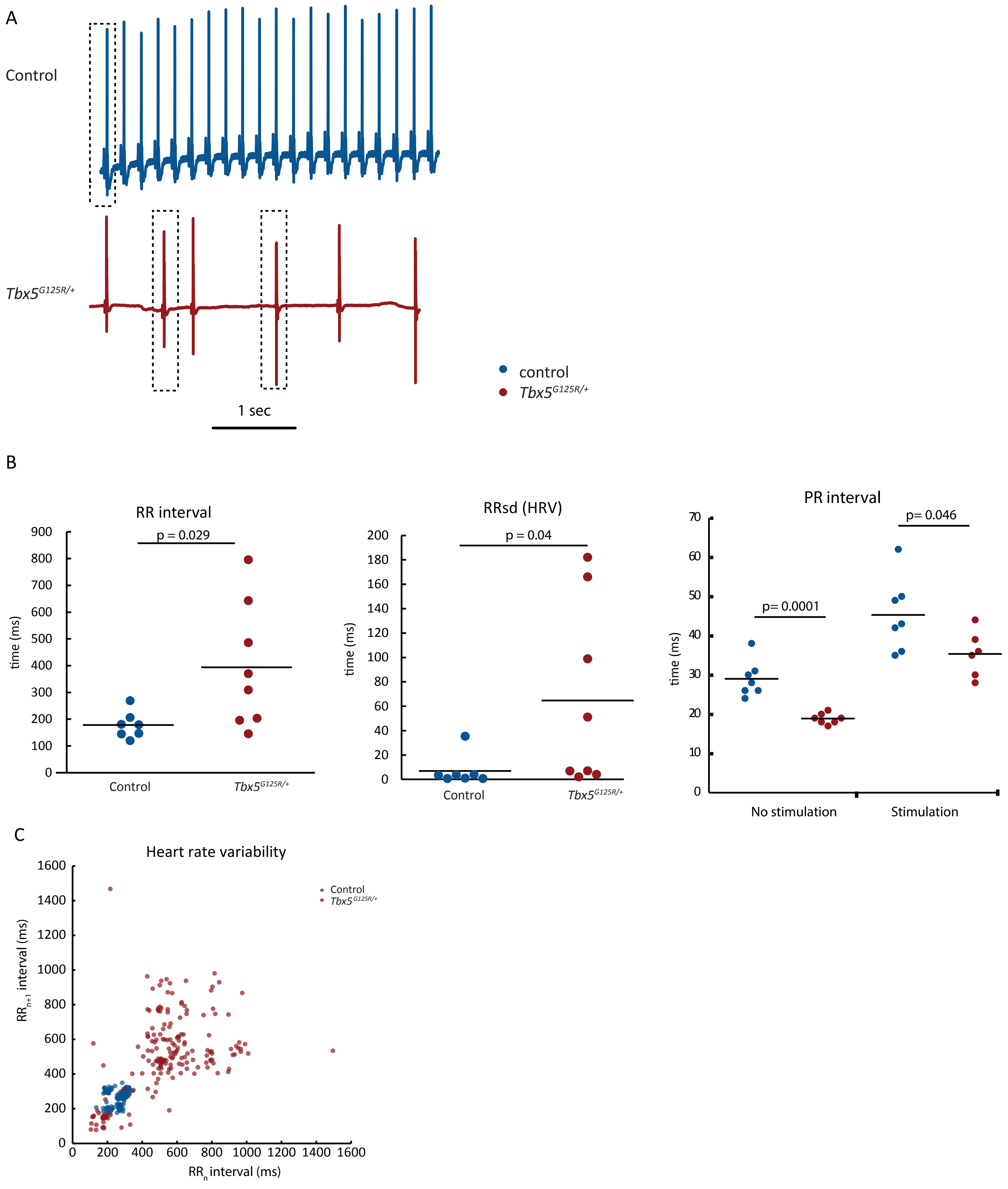

Supplemental Figure 4

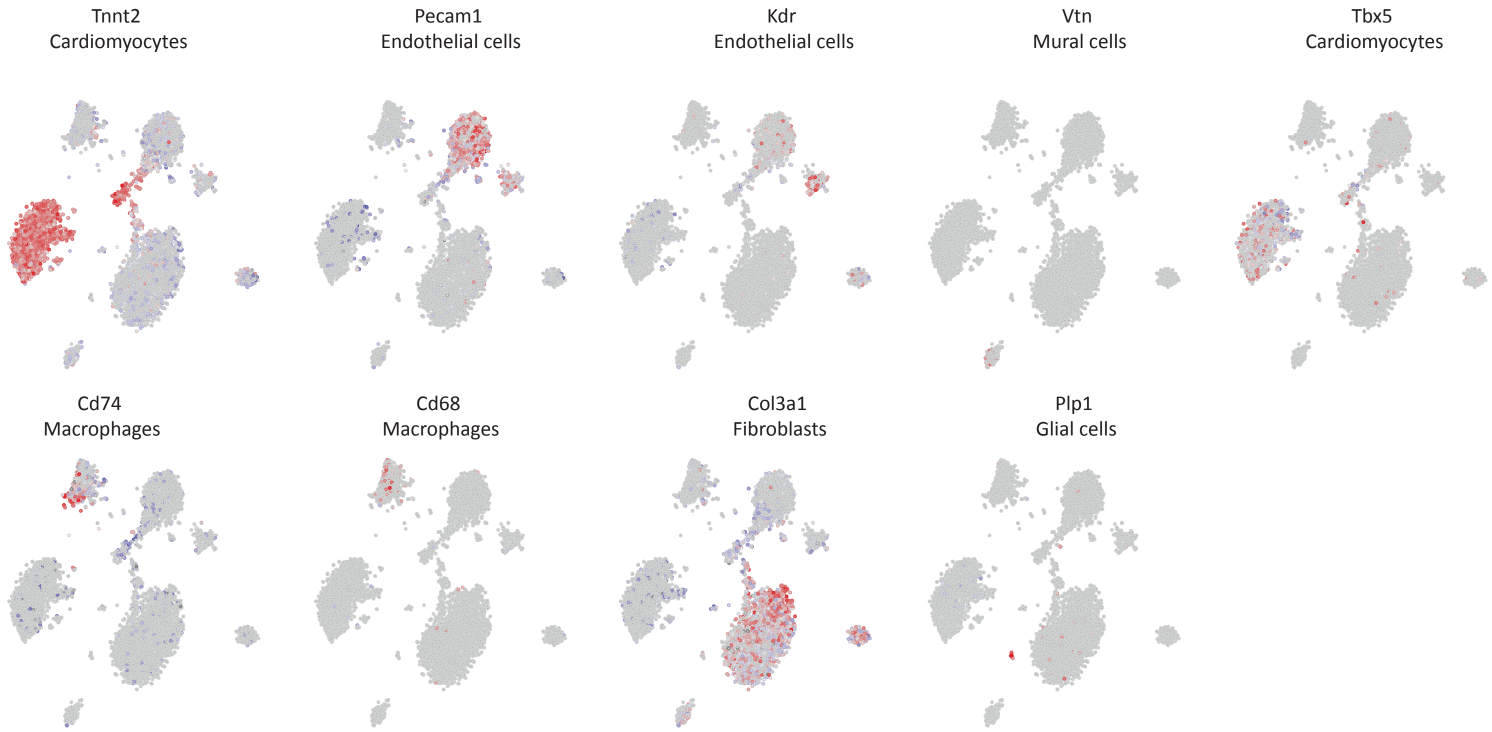

Supplemental Figure 5

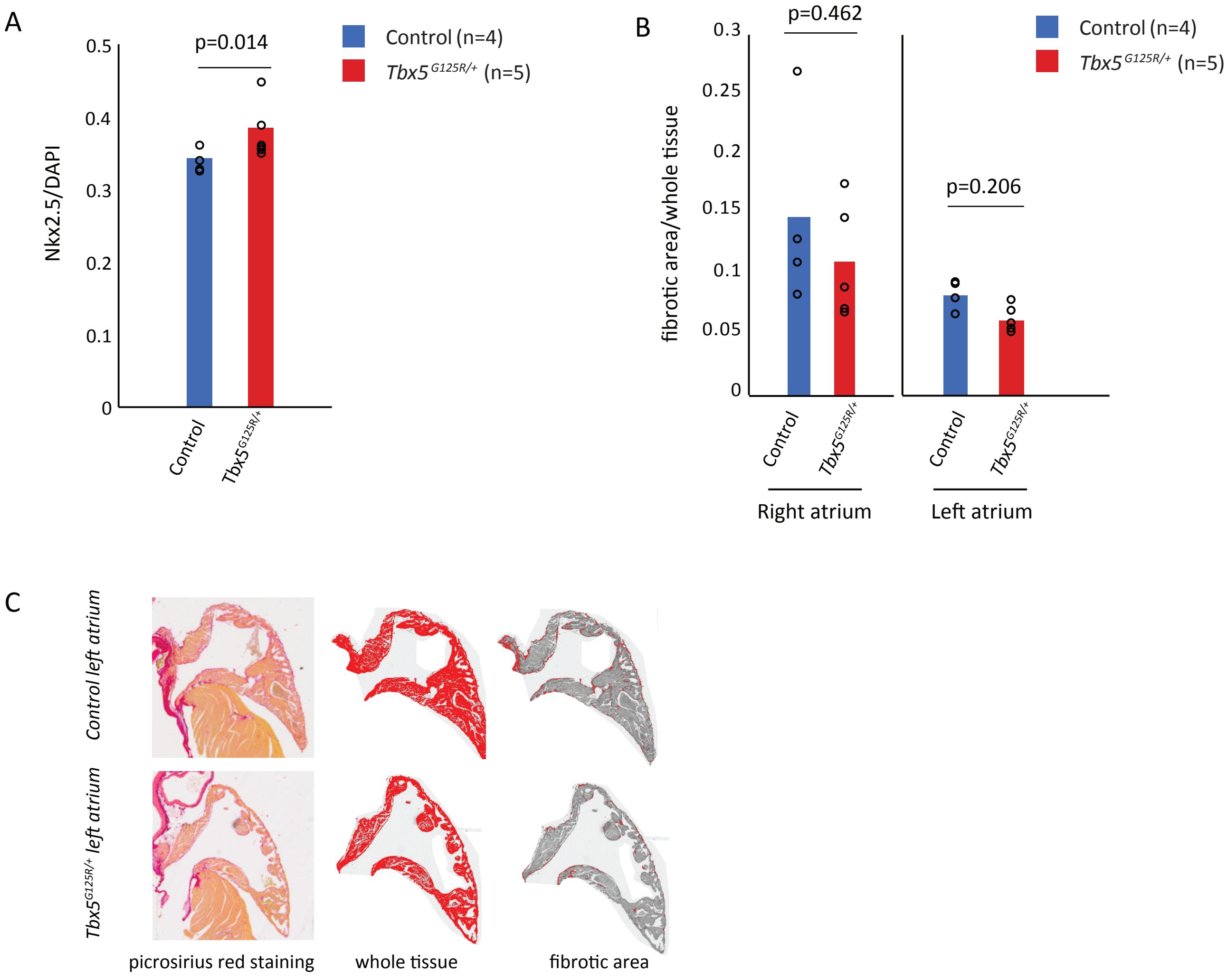

Supplemental Figure 6

A

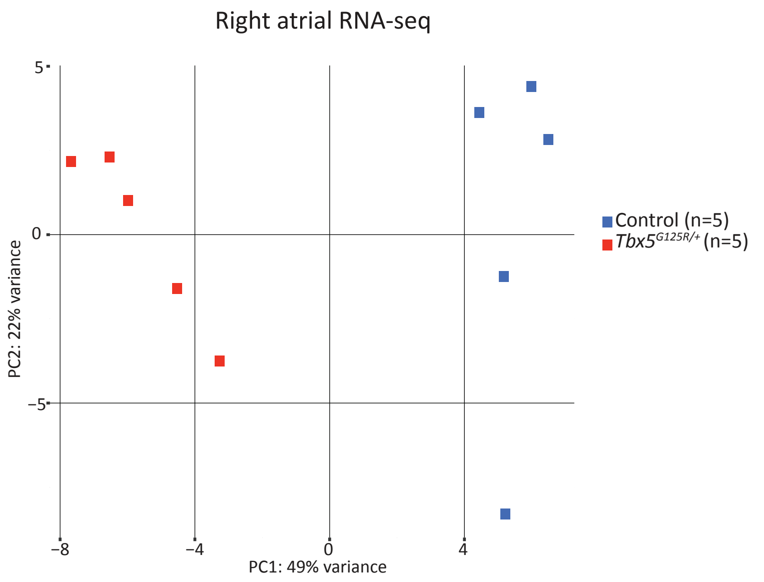

B

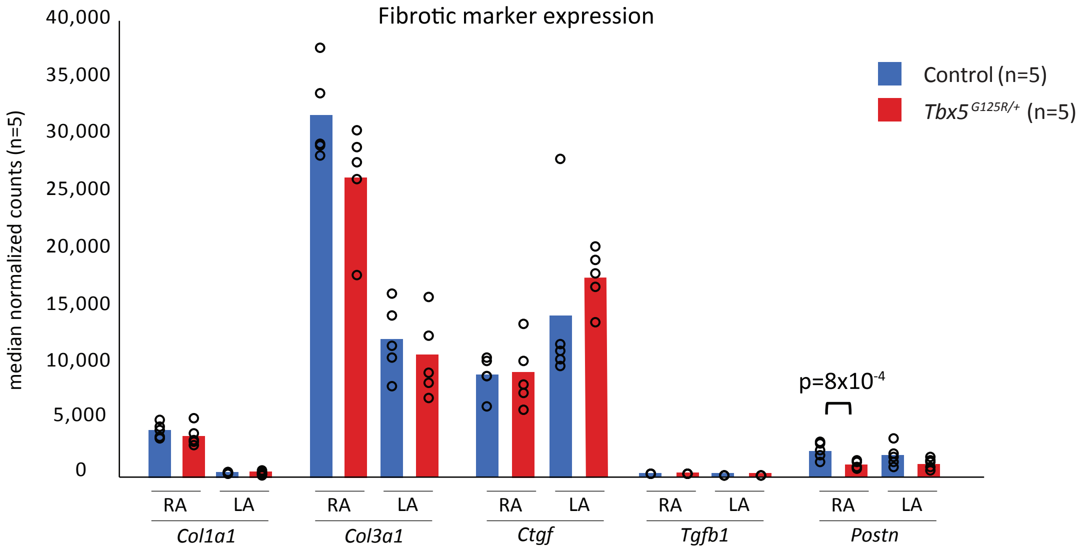

Supplemental Figure 7

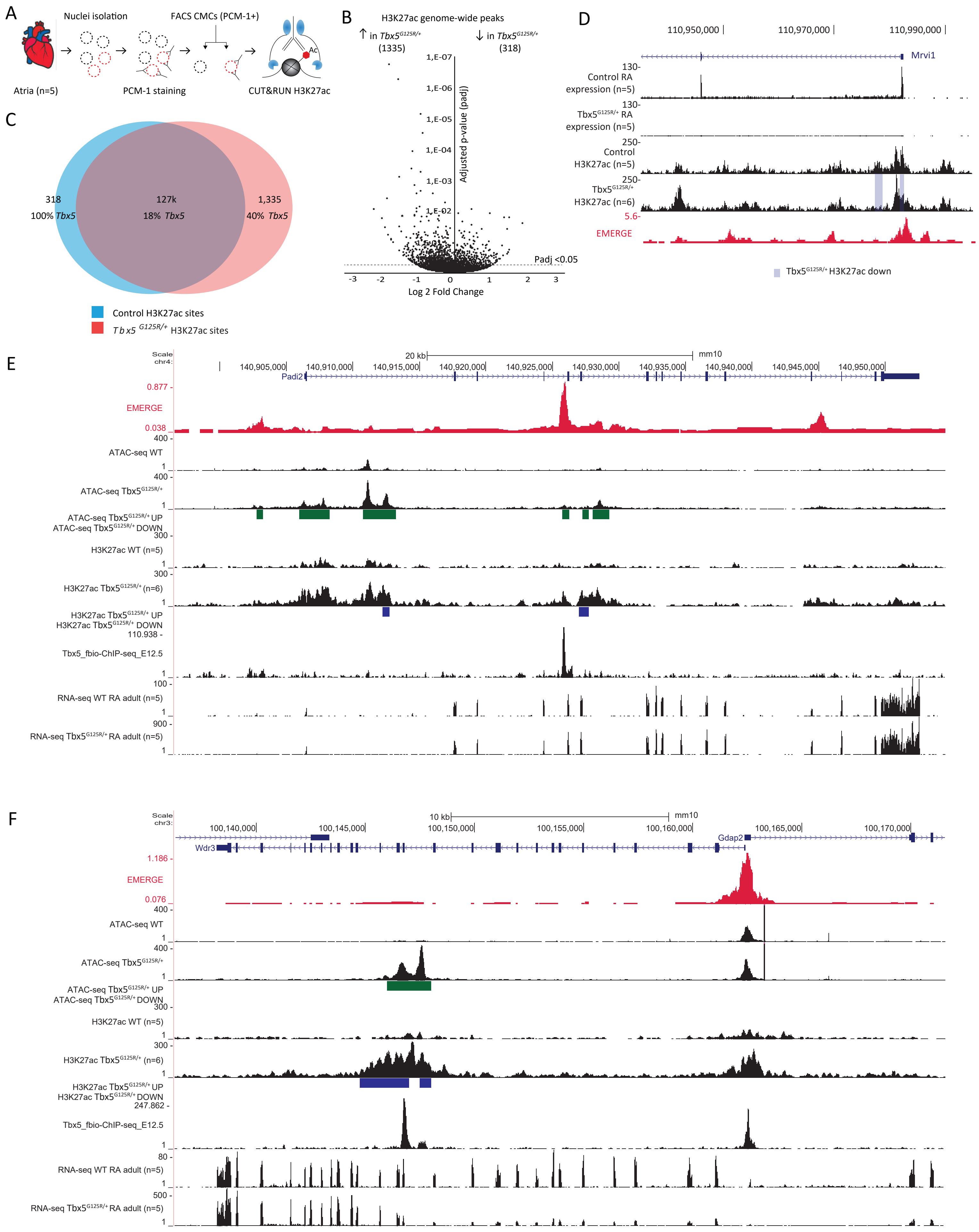

Supplemental Figure 8

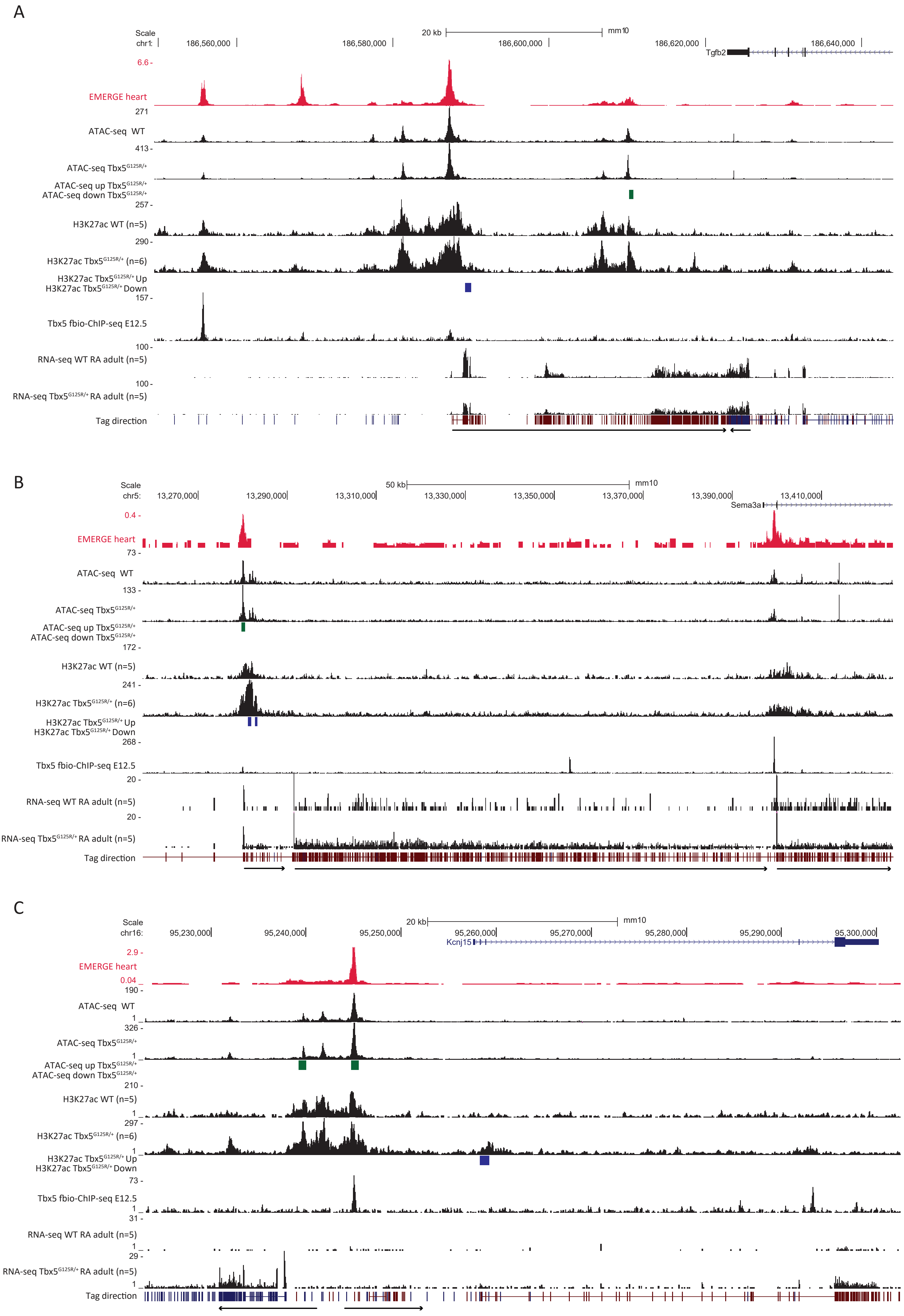

Supplemental Figure 9

A

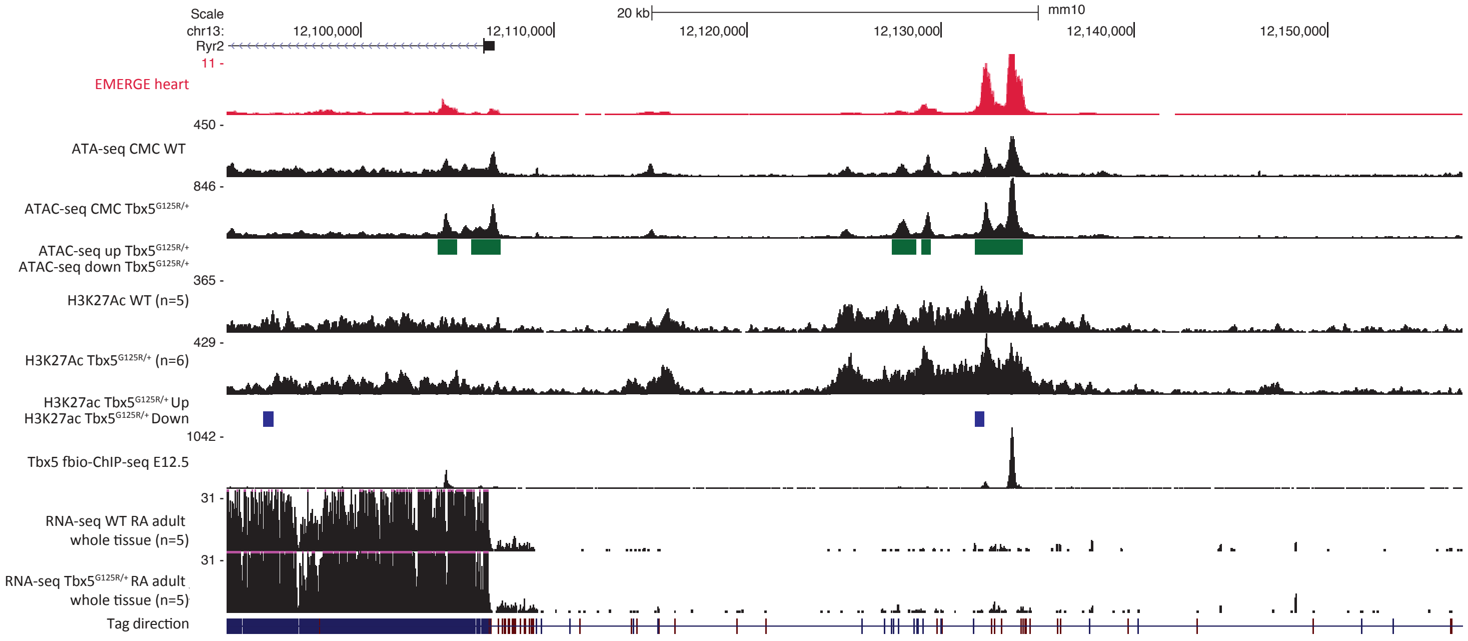

B

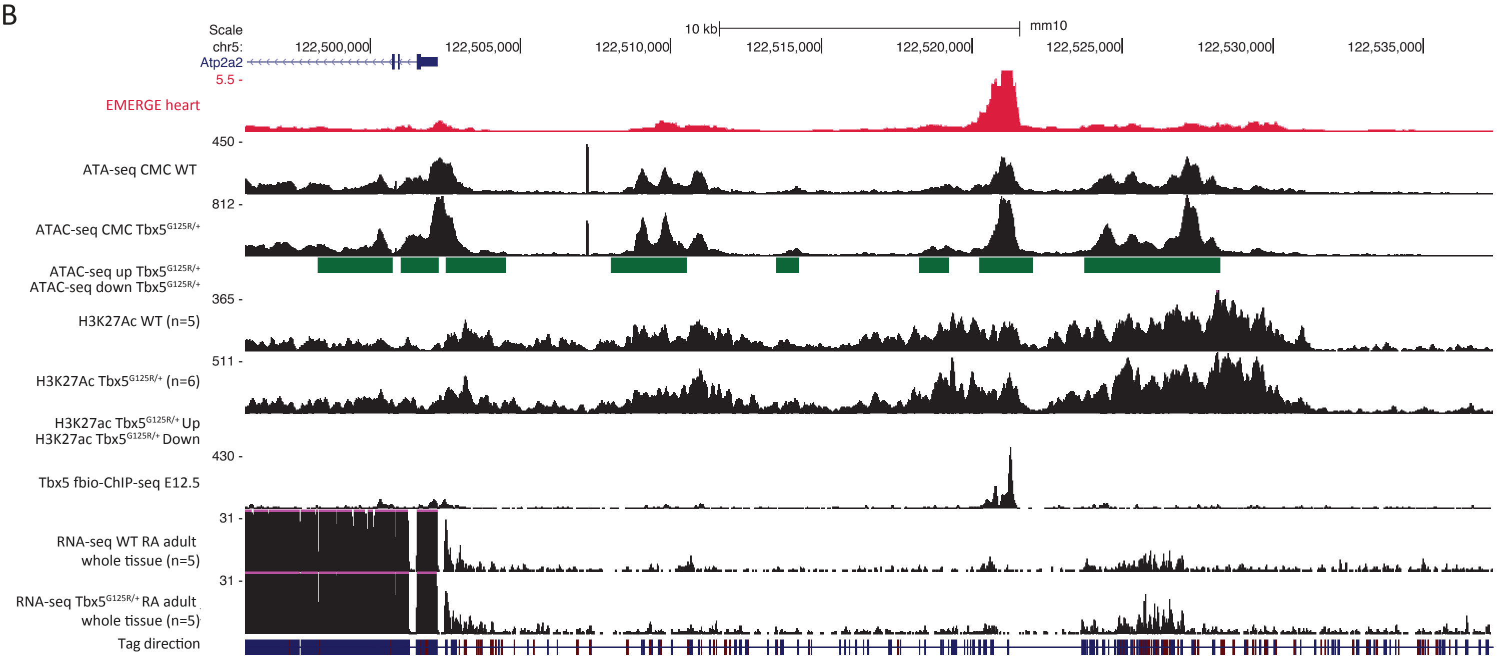

C

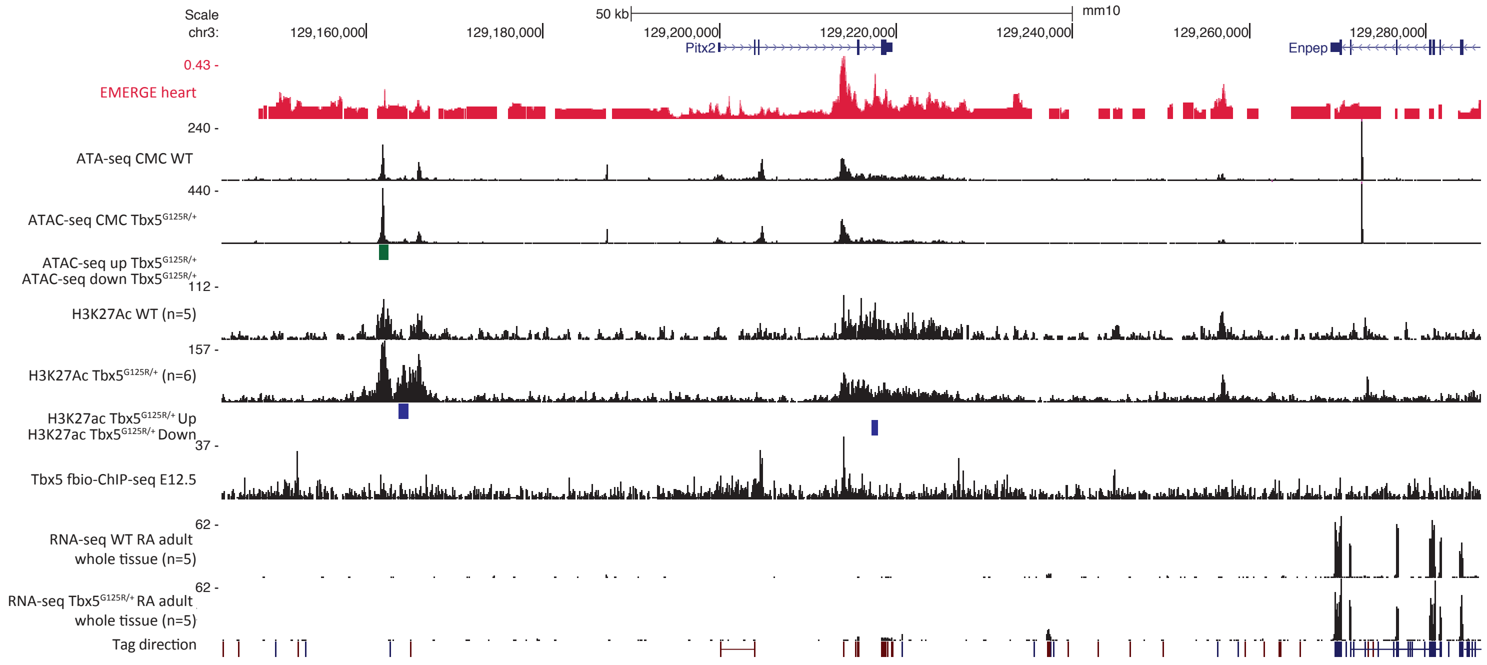

Supplemental Figure Legends

Supplemental Figure 1. Tbx5 pathological variant p.G125R leads to septal defects in homozygous animals, but no observable difference in spatial *Tbx5* expression in *Tbx5*$^{G125R/+}$ embryos. A) Morphological septal defects found in a E16.5 *Tbx5*$^{G125R/G125R}$ embryo. *Tbx5* whole mount *in situ* hybridization of control B) and C) *Tbx5*$^{G125R/+}$ E10.5 embryo both show characteristic *Tbx5* expression in the region above the eye (1), the ventricles (2), the atria (3), the front limbs (4), gonads (5). Abbreviations: E, embryonic developmental day.

Supplemental Figure 2. The proportion of proliferating cardio myocytes does not differ between control (n=5), *Tbx5*$^{G125R/+}$ (n=4) and *Tbx5*$^{G125R/G125R}$ (n=5) as shown by *Nkx2.5* and EdU staining in RA or LV in E12.5 embryos and expressed as the fraction dividing cardio myocytes (ratio Edu / Nkx2.5) (One-way ANOVA between genotypes per tissue type). Abbreviations: RA, right atrium; LV, left ventricle; E, embryonic developmental day.

Supplemental Figure 3. Langendorff ectopic action potential recording. A) Typical ECG traces of ex vivo control and *Tbx5*$^{G125R/+}$ hearts. B&C) Similar to the situation *in vivo,* hearts isolated from *Tbx5*$^{G125R/+}$ mice (n=8) showed longer and more variable RR intervals as well as shorter PR intervals than controls (n=8). (Mann Whitney U test). Abbreviations: RRsd, standard deviation of the difference between sequential RR intervals (RRn-RRn+1).

Supplemental Figure 4. Single nucleus (sn)RNA-seq T-distributed stochastic neighbor embedding (tSNE) map of cell-type specific marker gene expression.

Supplemental Figure 5. Cardiomyocyte and fibrosis in in right and left atria. A) Fraction of CMs (Nkx2.5) versus all cells (DAPI) differs between genotypes (Mann-Whitney U test). B) Measurement of the area of Picrosirius Red staining (fibrotic tissue) and the total tissue area, showed that in both RA and LA the median fraction of fibrotic tissue did not differ significantly. The genotype difference between ratio of fibrotic and whole tissue average was separately tested for the RA and LA with a Mann Whitney U test, showing a non-significant difference of a factor 2 between median values of fibrotic tissue in *Tbx5*$^{G125R/+}$ mouse atria of 8-month-old animals. C) Typical examples of Picrosirius Red staining of the LA of *Tbx5*$^{G125R/+}$ and control mouse.

Supplemental Figure 6. A) PCA plot of control (n=5) and *Tbx5*$^{G125R/+}$ (n=5) right atrial samples performed by the DESeq2 package. Abbreviations: PC(A), principle component (analysis). B) Fibrotic marker gene

expression of whole tissue RNA-seq of LA and RA shows only *Postn* marker gene is increased in expression in control mouse RA. Differential expression analysis on whole tissue RNA-seq was performed using the DESeq2 package based on a model using the negative binomial distribution. P-values were corrected for multiple testing using the false discovery rate (FDR) with 0.05 as control level.

Supplemental Figure 7. H3K27ac analysis using CUT&RUN. A) Method of CM nuclei isolation and H3K27Ac analysis. B) Scatter plot of H3K27ac in *Tbx5*$^{G125R/+}$ mouse atrium showing 1,335 sites with significantly increased acetylation and 318 with significantly decreased acetylation in the *Tbx5*$^{G125R/+}$ mouse atrium. Differential acetylation was assessed using the DESeq2 package based on a model using the negative binomial distribution P-values were corrected for multiple testing using the false discovery rate (FDR) method of Benjamini-Hochberg. C) Genome-wide H3K27ac sites in *Tbx5*$^{G125R/+}$ and control mouse atrium as well as overlap (in %) with known *Tbx5* binding sites[24]. Differences between relative cell population sizes overlapping *Tbx5* binding sites were determined using a Z-test. D) UCSC track showing *Mrvi1* decreased expression and decreased H3K27ac surrounding the promoter in *Tbx5*$^{G125R/+}$. Genome browser (UCSC) views of differentially accessible and H3K27ac associated regulatory elements in *Padi2* (E) and *Wdr3* (F). Tracks shown are predicted regulatory regions (EMERGE), (differential) accessibility (ATAC-seq), (differential) H3K27ac, Tbx5 binding in the fetal mouse heart and RNA expression in adult RA tissue of *Tbx5*$^{G125R/+}$ and control mice. Abbreviations: CUT&RUN, cleavage under targets, release under nuclease; PCM1, pericentriolar material-1; FACS, fluorescence-activated cell sorting.

Supplemental Figure 8. Genome browser (UCSC) views of differentially expressed ncRNAs identified near *Tgfb2* (A), *Sema3a* (B), *Kcnj15* (C). Tracks shown are predicted regulatory regions (EMERGE), (differential) accessibility (ATAC-seq), (differential) H3K27ac, *Tbx5* binding in the fetal mouse heart[24] and expression in RA whole adult tissue as well was tag direction of the transcription in *Tbx5*$^{G125R/+}$ and control mice. Tag direction of the transcription in RA is depicted in blue and red as well as arrows in the last two tracks.

Supplemental Figure 9. Genome browser (UCSC) views of loci of previously identified Tbx5-responsive ncRNAs[10, 12] near *Ryr2* (A), *Atp2a2* (B) and *Pitx2* (C) which are not responsive in *Tbx5*$^{G125R/+}$ mouse atria. Tracks shown ate predicted regulatory regions (EMERGE), (differential) accessibility (ATAC-seq), (differential) H3K27ac, *Tbx5* binding in the fetal mouse heart[24] and expression in RA whole adult tissue as well was tag direction of the transcription in *Tbx5*$^{G125R/+}$ and control mice. Tag direction of the transcription in RA is depicted in the last track in blue and red.
